# Simultaneous Ablation of the Catalytic AMPK α-Subunit SNF1 and Mitochondrial Matrix Protease CLPP Results in Pronounced Lifespan Extension

**DOI:** 10.3389/fcell.2021.616520

**Published:** 2021-03-04

**Authors:** Daniela Heinz, Evgeniia Krotova, Andrea Hamann, Heinz D. Osiewacz

**Affiliations:** Institute of Molecular Biosciences, J.W. Goethe University, Frankfurt am Main, Germany

**Keywords:** *Podospora anserina*, CLP protease, SNF1, AMPK, RMP1, aging, development

## Abstract

Organismic aging is known to be controlled by genetic and environmental traits. Pathways involved in the control of cellular metabolism play a crucial role. Previously, we identified a role of PaCLPP, a mitochondrial matrix protease, in the control of the mitochondrial energy metabolism, aging, and lifespan of the fungal aging model *Podospora anserina*. Most surprisingly, we made the counterintuitive observation that the ablation of this component of the mitochondrial quality control network leads to lifespan extension. In the current study, we investigated the role of energy metabolism of *P. anserina*. An age-dependent metabolome analysis of the wild type and a *PaClpP* deletion strain verified differences and changes of various metabolites in cultures of the *PaClpP* mutant and the wild type. Based on these data, we generated and analyzed a *PaSnf1* deletion mutant and a Δ*PaSnf1*/Δ*PaClpP* double mutant. In both mutants PaSNF1, the catalytic α-subunit of AMP-activated protein kinase (AMPK) is ablated. PaSNF1 was found to be required for the development of fruiting bodies and ascospores and the progeny of sexual reproduction of this ascomycete and impact mitochondrial dynamics and autophagy. Most interestingly, while the single *PaSnf1* deletion mutant is characterized by a slight lifespan increase, simultaneous deletion of *PaSnf1* and *PaClpP* leads to a pronounced lifespan extension. This synergistic effect is strongly reinforced in the presence of the mating-type “minus”-linked allele of the *rmp1* gene. Compared to the wild type, culture temperature of 35°C instead of the standard laboratory temperature of 27°C leads to a short-lived phenotype of the Δ*PaSnf1*/Δ*PaClpP* double mutant. Overall, our study provides novel evidence for complex interactions of different molecular pathways involved in mitochondrial quality control, gene expression, and energy metabolism in the control of organismic aging.

## Introduction

Aging of biological systems is characterized by a time-dependent decrease of physiological functions and an increase in morbidity and mortality. It has a multifactorial basis, and various molecular pathways are known to control aging, lifespan, and healthspan. Over decades of research, a paramount role of mitochondria, the cellular “power plants,” was repeatedly demonstrated in various systems from fungi to humans (Linnane et al., 1989; Cortopassi and Arnheim, 1990; Wallace, 1992; Melov et al., 1994; Petersen et al., 2003; Osiewacz, 2010; Breitenbach et al., 2012). In general, mitochondrial function declines during aging, although a number of different molecular pathways are active in keeping a “healthy population” of mitochondria over time (Luce et al., 2010; Baker et al., 2011; Fischer et al., 2012).

One of the aging model organisms in which the role of mitochondria was extensively investigated is the filamentous fungus *Podospora anserina* (Osiewacz, 2002; Scheckhuber and Osiewacz, 2008; Osiewacz et al., 2013). Classical genetic experiments identified an extrachromosomal genetic basis of aging already in the pre-molecular era (Marcou, 1962). Subsequently, this basis was located in mitochondria (Stahl et al., 1978; Cummings et al., 1979). Specifically, it was shown that the mitochondrial DNA (mtDNA) grossly reorganizes during the aging process as a result of the activity of a mobile DNA species that acts as a mutator (Kück et al., 1981, 1985; Osiewacz and Esser, 1984; Sellem et al., 1993; Borghouts et al., 2002). Later on, mitochondrial metabolism via the generation of adenosine triphosphate (ATP) at the inner mitochondrial membrane and the formation of reactive oxygen species (ROS) (Borghouts et al., 1997, 2001; Gredilla et al., 2006) was found to strongly affect the aging process. In more recent years, it was established that various pathways which are involved in the control of functional mitochondria have a strong impact on lifespan. For instance, it was shown that the overexpression of the gene coding for the mitochondrial LON protease leads to a healthspan extension via the increased degradation of damaged (carbonylated) proteins and the conservation of mitochondrial function (Luce and Osiewacz, 2009). While such an effect was expected, results from some other experimental interventions were counterintuitive and unexpected. For instance, the deletion of the gene coding for PaIAP, an AAA protease (ATPases associated with diverse cellular activities) that is located in the inner mitochondrial membrane, was found to lead to a lifespan extension (Weil et al., 2011). The molecular analysis revealed that PaIAP is a protease which, at controlled growth temperature of 27°C, is rather lifespan limiting. In contrast, at increased temperatures, as they are regularly reached during the day under natural conditions, the protease has a protective function. Yet another protease is PaCLPP which, together with the chaperone PaCLPX, is part of a multiprotein PaCLPXP complex located in the mitochondrial matrix. Deletion of the genes coding for both components of this complex resulted in healthspan extension of the corresponding mutants (Fischer et al., 2013; Knuppertz and Osiewacz, 2017). A detailed analysis of PaCLPXP interaction partners identified 47 proteins among which 19 turned out to be high confident substrates (Fischer et al., 2015b). Most of the substrates, including components of the pyruvate dehydrogenase complex, the tricarboxylic acid (TCA) cycle, and the respiratory chain, are involved in mitochondrial energy metabolism. Among these, there are components of the N-module of respiratory complex I which were subsequently also identified in mammals and the plant *Arabidopsis thaliana* (Szczepanowska et al., 2016; Petereit et al., 2020) suggesting at least a partial conserved role of CLPXP in pathways involved in cellular energy metabolism.

Initial data from studies with *P. anserina* revealed that ablation of the two components of the PaCLPXP protein complex does not affect cellular ATP levels most probably due to the conservation of cellular energy resulting from the induction of autophagy (Knuppertz and Osiewacz, 2017), a process that is stringently controlled by the nutrient status. In eukaryotes, two protein complexes, “target of rapamycin” (TOR) and “AMP-activated protein kinase” (AMPK), are master regulators of autophagy. The two complexes respond inversely to the cellular energy status. Under ATP-replete conditions, TOR is active and promotes anabolic processes as well as transcription and translation while inhibiting autophagy. Upon ATP depletion and AMP increase, AMPK becomes activated and stimulates various catabolic processes including autophagy (Knuppertz and Osiewacz, 2016; Wen and Klionsky, 2016; Yin et al., 2016).

Here we report an experimental study aimed to shed more light on metabolic aspects linked to the role of PaCLPXP in development and aging. We performed an age-related metabolome analysis of the wild type and the *PaClpP* deletion strain. Based on the results of this analysis and because of its role in autophagy induction, we investigated the consequences on aging of the ablation of the catalytic AMPK α-subunit (PaSNF1) in the wild-type and Δ*PaClpP* background. Surprisingly, we found that AMPK activity is not required for the lifespan extension of Δ*PaClpP* strains. Even more, lifespan extension of the *PaClpP* deletion mutant appears to be limited by AMPK. Most strikingly, we also observed a strong impact of the mating type, most likely the mating type-linked *rmp1* gene of *P. anserina*, on lifespan in a mutant in which *PaSnf1* and *PaClpP* are both deleted. These observations link protein quality control and the control of cellular energy with this rather poorly characterized RMP1 protein of *P. anserina*.

## Materials and Methods

### *P. anserina* Strains and Cultivation

The *P. anserina* wild-type (WT) strain “s” (Rizet, 1953), the previously described reporter strain *PaSod1::Gfp* (Zintel et al., 2010), the *PaClpP* deletion strain (Δ*PaClpP*; Luce and Osiewacz, 2009), and the following recently generated strains were used: Δ*PaSnf1*, Δ*PaSnf1*/*Flag::PaSnf1*, Δ*PaSnf1*/*PaSod1::Gfp*, Δ*PaSnf1*/Δ*PaClpP*, and Δ*PaSnf1*/Δ*PaClpP*/*PaSod1::Gfp*. Double mutants were obtained by crossing single-mutant strains. The triple-mutant Δ*PaSnf1*/Δ*PaClpP*/*PaSod1::Gfp* was obtained by crossing of Δ*PaSnf1*/Δ*PaClpP* with *PaSod1::Gfp*. All mutant strains were constructed in the genetic background of the WT “s.” Unless otherwise stated, strains were grown on complete medium (BMM) containing cornmeal extract (Esser, 1974). To induce the formation of fruiting bodies, cultures with opposite mating types were placed on solid semi-synthetic M2 agar plates (M2) (Osiewacz et al., 2013) and incubated for 10–14 days at 27°C and constant light. For spore germination, the spores were incubated for 2 days at 27°C in the dark on BMM containing 60 mM ammonium acetate. Strains were grown on M2 (0.025% KH_2_PO_4_, 0.03% K_2_HPO_4_, 0.025% MgSO_4_, 0.05% urea, 1% dextrin, 2% agar), M2 + glycerol (0.025% KH_2_PO_4_, 0.03% K_2_HPO_4_, 0.025% MgSO_4_, 0.05% urea, 1% glycerol, 2% agar), or M2-N (0.025% KH_2_PO_4_, 0.03% K_2_HPO_4_, 0.025% MgSO_4_, 1.5% dextrin, 2% agar) or in liquid complete medium [CM: 0.1% KH_2_PO_4_, 0.05% KCl, 0.05% MgSO_4_, 1% glucose, 0.37% NH_4_Cl, 0.2% tryptone, 0.2% yeast extract, 0.1% stock solution A (0.1% ZnSO_4_, 0.1% FeCl_2_, 0.1% MnCl_2_, pH 6.5 (KOH)), pH 6.5 (KOH)] at 27°C under constant light (Osiewacz et al., 2013).

### Metabolic Profiling

To analyze putative changes in the abundance of metabolites in the *PaClpP* deletion mutant compared to the wild type, a metabolomic profiling was performed with 5- and 20-day-old wild-type and Δ*PaClpP* strains (*n* = 4 each). Mycelial extracts were obtained from strains grown on M2 agar plates at 27°C and constant light for a specific time. Subsequently, the mycelium of the aged strains (17-day-old strains) and young strains (2-day-old strains, directly used from the BMM plates with ammonium acetate) were inoculated on M2 agar plates, covered with cellophane, and allowed to overgrow the plates for three additional days at 27°C and constant light. The agar pieces were removed, the mycelium was harvested and portioned in 50-mg aliquots into a 1.5-ml tube. The samples were frozen in liquid nitrogen and stored at −80°C until the final analysis. The analysis was performed by Metabolomic Discoveries (Metabolomic Discoveries GmbH, Potsdam, Germany). The used optimized method allows the unambiguous characterization and relative quantification of the requested metabolites. Metabolite analysis by LC–tandem mass spectrometry was performed using a LCMS-8050 by Shimadzu triple-quadrupole mass spectrometer equipped with an electrospray ionization (ESI) source and operated in the multiple reaction mode (MRM).

### Cloning Procedures and Generation of *P. anserina* Mutants

All strains were constructed in the genetic background of the wild-type strain “s” (Rizet, 1953). To generate the Δ*PaSnf1* strain, we proceeded as described by Hamann et al. (2005). First, the flanking regions of *PaSnf1* were amplified with the following oligonucleotides: For the 5′-flanking region, Snf1KO1 (CAAGGGCACGGTGCTGTC, Biomers, Ulm, Germany) and Snf1KO2 (TTAAGCTTCATGCTGGCGACGGCTATC, HindIII site is underlined, Biomers, Ulm, Germany) were used. For the 3′-flank, Snf1KO3 (CGCGACTAGTGCGGATGCGGA TTGATTATTG, BcuI site is underlined, Biomers, Ulm, Germany) and Snf1KO4 (AAGCGGCCGCTGTCGATGATA CAGATAG, NotI site is underlined, Biomers, Ulm, Germany) were used. These amplified flanking regions were cloned in the plasmid pKO4 (Hamann et al., 2005; Luce and Osiewacz, 2009), using the restriction enzymes KpnI, BcuI, NotI, and HindIII (Thermo Scientific, Waltham, MA, United States, ER0521, ER1251, ER0591, ER0501; KpnI site is part of the 5′-flank and thus has not to be added through the oligonucleotide). The wild-type *PaSnf1* gene in a cosmid selected from a representative cosmid library (Osiewacz, 1994) was replaced in *Escherichia coli* by a combined blasticidin/phleomycin resistance cassette as described by Hamann et al. (2005). The resulting replacement cosmid was used to transform *P. anserina* wild-type spheroplasts (Osiewacz et al., 1991). Transformants were selected on a phleomycin- (Genaxxon, Ulm, Germany, M3429) containing medium. For the complementation of the *PaSnf1* deletion mutant, and additionally to mark the PaSNF1 protein with a His::FLAG epitope, the sequence of *PaSnf1* was fused with the sequence encoding this epitope. To achieve this, the sequence encoding the His::FLAG epitope was introduced in the plasmid pKO7 (Kunstmann and Osiewacz, 2009), which contains a *hygromycin* B resistance cassette. To include the *PaSnf1* gene in this pHisFLAG vector, the gene was amplified in two parts. The first part consists of the promoter region, which was amplified with the oligonucleotides Snf1-4 (AAGCGGCCGCCATGGAAATAAGGACCCAG, NotI site is underlined) and Snf1-11 (TTGGATCCCATGCTGGCGACG GCTAT, BamHI site is underlined). The second part contains the *PaSnf1* ORF and 500 bp of the terminator region, which were amplified with the oligonucleotides Snf1-12 (ATGGCCCAGGCCTACGACGACGA, this oligonucleotide contains a phosphate residue at the 5′-ending) and Snf1-9 (ACCTGCAGTGCCAGAATGGCCAGCCTTATG, PstI site is underlined). The PCR product of the *PaSnf1* ORF and the pHisFLAG vector was digested with the restriction enzymes SmaI and PstI (Thermo Scientific, Waltham, MA, United States, ER0661, ER0611). The constructed vector pHis-Flag::PaSnf1-1 contains the *PaSnf1* ORF fused with a sequence encoding a HIS::FLAG epitope at the 5′-ending. In a second step, the promoter region of *PaSnf1* was integrated into the newly constructed plasmid pHis-Flag::PaSnf1-1 using the restriction enzyme sites of NotI and BamHI (Thermo Scientific, Waltham, MA, United States, ER0591, ER0051). The final plasmid pFlag-Snf1 contains the sequence of a HIS::FLAG epitope between the sequence of the promoter region of *PaSnf1* and the *PaSnf1* ORF with the terminator region and was transformed into *P. anserina* wild-type spheroplasts as previously described (Osiewacz et al., 1991), and hygromycin B [Merck Millipore (Calbiochem), Burlington, MA, United States, 400051]-resistant transformants were selected. Finally, the newly generated strain *Flag::PaSnf1* was crossed with the *PaSnf1* deletion mutant to remove the endogenous *PaSnf1* sequence. The new Δ*PaSnf1*/*Flag::PaSnf1* strain was selected by the phleomycin and hygromycin B resistance and verified by Southern blot analysis.

### Southern Blot Analysis

Total DNA was isolated as described before (Lecellier and Silar, 1994). Restriction of the DNA, gel electrophoresis, and Southern blotting were performed in accordance with standard protocols (Sambrook et al., 1989). Hybridization and detection were accomplished as described in the manufacturer’s protocol with digoxigenin-labeled hybridization probes (DIG DNA Labeling Mix, Sigma-Aldrich, St. Louis, MO, United States, 11175033910). The *PaSnf1*-specific probe was generated by PCR with the oligonucleotides Snf1-1 (GCATCGGCGCCTACAACATTGTC, Biomers, Ulm, Germany) and Snf1-2 (GAGACCGAAATCGGCGATCTTG, Biomers, Ulm, Germany). The *phleomycin* (*Ble*)-specific probe was generated from the plasmid pKO4 (Luce and Osiewacz, 2009), which contains the phleomycin resistance cassette. The respective fragment was recovered after digestion with BamHI. The resulting 1.3-kb fragment was extracted and purified from the agarose gel. The *PaClpP*-specific probe was generated according to Fischer et al. (2013).

### Quantitative Real-Time PCR (qRT-PCR)

For isolation of total RNA, strains were allowed to overgrow a cellophane foil-covered M2 petri dish for 3 days at 27°C and constant light. The mycelium was subsequently transferred into 2-ml tubes with 0.5-mm glass beads (Bertin Technologies, Montigny-le-Bretonneux, France, KT03961-1-004.2) and disrupted in a Precellys 24 homogenizer (Bertin Technologies; peqlab; Precellys 24 lysis and homogenization, Montigny-le-Bretonneux, France) for 2 × 25 s at 5800 rpm and a 10-s break in between. The isolation was performed using the NucleoSpin RNA Plant kit (Macherey Nagel #740949.250, Düren, Germany) according to the manufacturer’s instruction. Samples of wild type, Δ*PaSnf1*, Δ*PaClpP*, and Δ*PaSnf1*/Δ*PaClpP* strains were analyzed. 1 μg RNA was used for the reverse transcription with a Revert Aid Reverse transcriptase and Ribolock RNase inhibitor (Thermo Scientific, Waltham, MA, United States, EP0441, EC0381) and oligo(dT)_18_ (Thermo Scientific, SO132). 2 μl of the resulting cDNA was used for the quantitative real-time PCR (qRT-PCR) reaction (IQ Sybr Green SuperMix, Bio-Rad, Hercules, CA, United States). To determine the expression of *PaMdm38*, the primers Pa_1_1789-1 (GCCCAGAAGGAAGAGTTTAC) and Pa_1-1780-2 (GTTGGAGACACCATCACTAC) were used. For each gene, the PCR efficiency was determined according to Pfaffl (2001). The relative expression level was normalized to the expression of *PaPorin* which was determined with the oligonucleotides Porin-RT-for (TCTCCTCCGGCAGCCTTG, Biomers, Ulm, Germany) and Porin-RT-rev (CGGAGGCGGACTTGTGAC, Biomers, Ulm, Germany). The relative expression level was calculated with the following formula:

Relative expression = (E(porin)^CP(porin))/(E(target gene)^CP(target gene));

E = PCR efficiency of the respective primer pair;

CP = crossing point for each transcript.

### Growth Rate and Lifespan Analysis

The lifespan and growth rate of monokaryotic isolates at 27 and 35°C were determined using race tubes containing M2 medium as described previously (Osiewacz et al., 2013). Lifespan analyses on M2 with glycerol instead of dextrin, or on the autophagy-inducing M2 medium without the nitrogen source urea but with the 1.5-fold concentration of dextrin (M2-N), were performed in petri dishes. The petri dishes and the race tubes were incubated at 27 or 35°C and constant light. The lifespan is defined as the time period in days (d) of linear growth. The growth rate is defined as the growth in centimeters (cm) per day (d). For measurement, the growth front of the strains was marked every 2–3 days until death.

### Microscopy and Spore Analysis

To analyze the content of the fruiting bodies and the spore morphology, confrontational crosses of different strains were performed on M2 medium. The strains were placed 3 cm apart of each other and were incubated for 12 (wild type × wild type; wild type × Δ*PaSnf1*; Δ*PaSnf1*/*Flag::PaSnf1* × Δ*PaSnf1*; Δ*PaSnf1* × Δ*PaSnf1*) and 14 days (Δ*PaSnf1* × Δ*PaSnf1*) at 27°C and constant light. The content of a fruiting body was placed in a 20-μl H_2_O droplet on a slide and covered with a cover slip. The samples were observed with the Leica Biomed Microscope (Leica Biosystems Nussloch GmbH, Nußloch, Germany) and documented with the camera DFC7000 T. To analyze the fruiting body content, the 10×/0.25 objective lens (506088) was used. To visualize spore morphology, the 40×/0.65 objective lens (506099) was used. For quantification of fruiting body content, spores of 10 fruiting bodies of each cross were analyzed (wild type × wild type *n* = 1691 spores, Δ*PaSnf1* × Δ*PaSnf1*: *n* = 760 spores; wild type × Δ*PaSnf1*: *n* = 1595 spores; Δ*PaSnf1*/*Flag::PaSnf1* × Δ*PaSnf1*: *n* = 1423 spores).

### Fertility Analysis

Determination of male (♂) or female (♀) fertility was performed by spermatization experiments. Cultures derived from mononucleate ascospores of the wild type (*n* = 3 of each mating type) and Δ*PaSnf1* (*n* = 3 of mating type “minus” and *n* = 2 of mating type “plus”) were grown for 7 days on M2 agar plates at 27°C and constant light. To harvest spermatia, the mycelium was overlaid with 5 ml sterile water and incubated for 5 min. Afterward the water containing spermatia was collected from the plate. For spermatization, 300 μl of this suspension was dropped on mycelium of the opposite mating type (*n* = 5 technical replicates each). After 5 min of incubation, the droplets were removed and the plates were incubated for 5 days at 27°C and constant light. The number of perithecia obtained from wild-type crosses was set to 100%. For each case [wild type (♂) × wild type (♀); wild type (♂) × Δ*PaSnf1* (♀), Δ*PaSnf1* (♂) × wild type (♀); Δ*PaSnf1* (♂) × Δ*PaSnf1* (♀)], six different spermatizations were used for quantification.

### Fluorescence Microscopy

For this experiment, *P. anserina* strains were cultivated on glass slides with a central depression containing 160 μl M2 medium for 1 day at 27°C and constant light in a moisture-prone chamber. For microscopic analysis, the Zeiss Cell Observer SD-Spinning Disk Confocal Microscope (Carl Zeiss Microscopy, Jena, Germany) with a 100×/1.46 oil objective lens (Carl Zeiss Microscopy, Jena, Germany, 420792-9800) was used. For visualization of mitochondria, the hyphae were stained with 100 μl of 2 μM MitoTracker Red (Invitrogen, Carlsbad, CA, United States) for 15 min in the dark. Subsequently, the hyphae were washed with 100 μl water. The mitochondria were visualized with the appropriate excitation and emission filters. For image processing, ZEN 2.5 (blue, Carl Zeiss Microscopy, Jena, Germany) was used.

### Measurement of Oxygen Consumption Rate

The measurement of oxygen consumption rate was performed at 27°C by high-resolution respirometry (Oxygraph-2k series C and G, OROBOROS Instruments, Innsbruck, Austria). Cultures derived from mononucleate ascospores of wild type, Δ*PaSnf1*, Δ*PaClpP*, and Δ*PaSnf1*/Δ*PaClpP* were grown on M2 plates covered with a cellophane foil for 2 days, shifted into CM liquid medium, and further incubated for 3 days at 27°C under shaking and constant light. A small piece of mycelium was placed into the Oroboros respirometer chamber into 2 ml fresh CM media. The oxygen consumption rate was determined as previously described in Fischer et al. (2015a). After the measurement, the mycelium was placed into a 2-ml tube and boiled for 10 min at 95°C. Subsequently, the mycelium was air-dried for 2 days, and dry weight was determined. Finally, basal oxygen consumption per mg mycelium was calculated. For the analysis of the data, the DatLab6 software from Oroboros (Innsbruck, Austria) was used.

### Western Blot Analysis

For isolation of total protein extracts, the strains were allowed to age on M2 plates at 27°C under constant light, after spore germination on BMM plates with 60 mM ammonium acetate for 2 days at 27°C in the dark. Mycelia from the different *P. anserina* strains and of different age were allowed to overgrow the cellophane foil-covered solid M2 medium for 3 days at 27°C and finally shifted to CM liquid medium and further incubated for 3 days at 27°C under constant light and shaking. To harvest the mycelium, the cultures were filtered over two layers of gauze. Approximately 250 mg mycelia was transferred in 2-ml tubes with 0.5-mm glass beads (Bertin Technologies, Montigny-le-Bretonneux, France, KT03961-1-004.2), and two volumes of protein extraction buffer (50 mM HEPES, 100 mM NaCl, 5 mM DTT, pH = 7.4) were added. The samples were kept on ice. For homogenization of the mycelia, the *Precellys* homogenizer (Bertin Technologies; peqlab; Precellys 24 lysis and homogenization, Montigny-le-Bretonneux, France) was used (2 × 25 s at 5800 rpm with a 10-s break in between). Afterward, the samples were centrifuged for 5 min at 9300 × *g* and 4°C. Subsequently, the supernatant was transferred to a new 1.5-ml reaction tube. The isolation of mitochondrial protein extracts was performed as previously described by Kunstmann and Osiewacz (2008). For western blot analysis, 50–150 μg protein extract was mixed with SDS-loading buffer (6% TRIS/HCl pH 6.8, 48% glycerol, 9% β-mercapto-ethanol, 0.03% bromophenol blue, 6% SDS) and heated up for 10 min at 95°C. The separation of proteins was accomplished by SDS-PAGE by standard protocols (Brust et al., 2010), and the proteins were subsequently transferred to a PVDF membrane (Immobilon-FL PVDF, Millipore, Burlington, MA, United States, IPFL00010). Blocking and antibody treatment of the membrane were performed according to protocols of the Odyssey^®^ Imaging System Western blot analysis (LI-COR Biosciences, Bad Homburg, Germany). The following primary antibodies were used for this study: anti-Phos-αAMPK (Phospho-AMPKα (Thr172) (40H9), rabbit mAb (dilution 1:300, Cell Signaling Technology, Danvers, MA, United States, #2535L), anti-FLAG (mouse, dilution 1:1,000, Sigma, F3165), anti-GFP (mouse, dilution 1:10,000, Sigma-Aldrich, St. Louis, MO, United States, G6795), and anti-PaDNM1 (rabbit, dilution 1:5,000, Sigma (NEP), polyclonal antibody raised against synthetic peptide “Ac-DKGTPEKESIAIRKC-amide,” affinity purification). IRDye^®^ 680RD anti-mouse (goat, dilution 1:15,000, LI-COR Biosciences, Bad Homburg, Germany, 926-68070) and IRDye^®^ 680LT anti-rabbit (goat, dilution 1:20,000, LI-COR Biosciences, Bad Homburg, Germany, 926-68021) were used as secondary antibodies.

### Statistical Analysis

The statistical analysis of lifespans was performed with the IBM SPSS statistics 19 software package, and a Kaplan–Meier survival estimate was generated. Significance was evaluated using three independent statistical tests (Breslow, generalized Wilcoxon; log rank, Mantel–Cox; Tarone-Ware). In the figure legends, only the lowest *p*-values are indicated. A table with the exact values can be found in the Supplementary Tables 2, 3. To determine the significance of the other experiments, two-tailed Student’s *t*-test was used (^∗∗∗^: *p* < 0.001; ^∗∗^: *p* < 0.01; ^∗^: *p* < 0.05).

## Results

### Ablation of PaCLPP Affects TCA Cycle, Glucose and Amino Acid Metabolism, and Nucleotide Levels

The previous demonstration of potential substrates of the mitochondrial PaCLPXP complex, the majority of which are associated with metabolic pathways in mitochondria (Fischer et al., 2015b), and the observation that the ablation of the protease and chaperone component of the complex leads to a pronounced lifespan extension (Fischer et al., 2013) directed our interest to more carefully investigate the impact of metabolic processes on aging of *P. anserina*.

In a first series of experiments, we investigated the metabolome of the Δ*PaClpP* mutant of 5- and 20-day-old cultures and compared it with that of the wild type of the same chronological age. In addition, the metabolomes of the wild type of 5- and 20-days of age were analyzed. At note, since the two strains are characterized by a different lifespan, their physiological status (i.e., their biological age) at a given chronological age is not identical. For instance, while 20-day-old wild-type strains are almost senescent and functionally impaired, Δ*PaClpP* mutant strains are rather unaffected.

**FIGURE 1:**
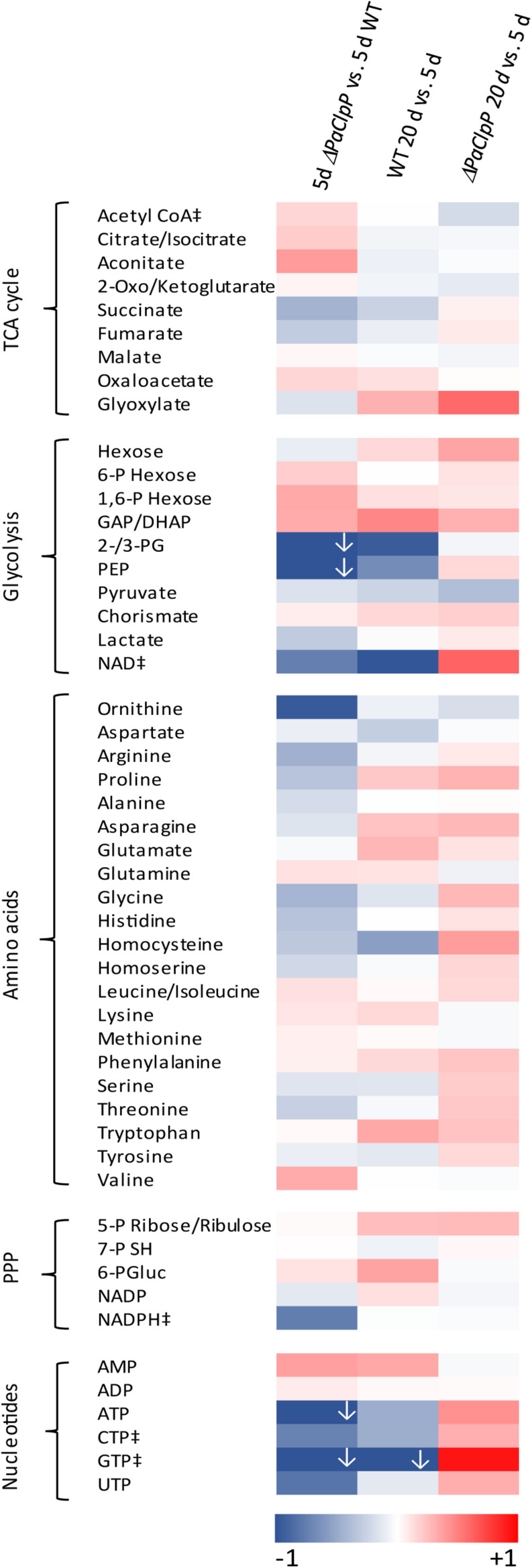
Deletion of *PaClpP* affects the energy metabolism. Color-coded heat map shows the fold change log(2) of 5-day-old Δ*PaClpP* vs. wild-type strains, 20-day-old wild-type vs. 5-day-old wild-type strains, and 20-day-old Δ*PaClpP* vs. 5-day-old Δ*PaClpP* strains of four biological replicates, each. For better visualization, log2-fold changes lower than 1 (indicated by ↓) are also presented in darkest blue. In Supplementary Table 1, the exact values are provided. “‡”: In at least one biological replicate, this metabolite is at the detection limit. Abbreviations: citrate/isocitrate, citrate or isocitrate; 2-oxo/ketoglutarate, 2-oxoglutarate or 2-ketoglutarate; 6-P hexose, hexose 6-phosphate; 1/6-P hexose, hexose 1,6-bisphosphate, GAP: glyceraldehyde 3-phosphate; DHAP, dihydroxyacetone phosphate; 2-/3-PG, 2- or 3-phosphoglycerate; PEP, phosphoenolpyruvate; 5-P ribose/ribulose, ribose 5-phosphate or ribulose 5-phosphate; 7-P SH, sedoheptulose 7-phosphate; 6-PGluc, 6-phosphogluconate; PPP, pentose phosphate pathway.

The metabolome analysis was performed with cell extracts isolated from mycelia of four biological replicates of each strain and age and the relative amount of 50 metabolites was determined by triple-quadrupole mass spectrometry. The complete data set is presented in Supplementary Table 1 and visualized in a heat map (Figure 1). Most striking alterations occur in the abundance of components of the TCA cycle, of nucleotides, glycolysis metabolites, and amino acids between young (5-day-old) mutant and wild-type cultures (Figure 1; left). Interestingly, some of these alterations are also observed in wild-type cultures of different age (Figure 1; WT 20 day vs. 5 day). In particular, the abundance of glycolysis metabolites and of nucleotides changes during aging in the same direction compared to the situation in young mutant vs. young wild-type strains. It appears that these alterations are rather not responsible for the observed lifespan extension of the mutant. In contrast, changes in amino acid and TCA cycle metabolite levels differ in the aging wild type compared to young mutant vs. young wild type. Especially a significant increase in citrate/isocitrate and aconitate levels is observed in the 5-day-old mutant compared to wild type of the same chronological age. No change in abundance of these metabolites is observed in wild-type cultures of 5 and 20 days of age. Moreover, the abundance of several amino acids (arginine, proline, glycine, histidine, and threonine) is significantly lower in 5-day-old mutants compared to wild types of the same chronological age but not in 20-day-old wild types compared to 5-day-old wild-type cultures. In addition, during wild-type aging several metabolite levels (especially glycolysis metabolites) significantly decreased, while during Δ*PaClpP* aging (20 days vs. 5 days, Figure 1; right) rather an overall increase in metabolite levels is observed. This effect may result from the upregulation of non-selective autophagy in the Δ*PaClpP* mutant that was observed in a previous study (Knuppertz and Osiewacz, 2017) as a compensatory response to mitochondrial impairments in the mutant. This idea is supported by the observed changes in the abundance of several nucleotides. Most interestingly, AMP is increased while ATP levels decrease in the young mutant compared to young wild type. This increase in the AMP/ATP ratio directed us to a potential induction of AMPK signaling in the Δ*PaClpP* mutant, which may explain its lifespan-extended phenotype. To address this possibility experimentally, we set out to delete the gene encoding the catalytic α-subunit of the AMPK complex, termed SNF1 in fungi, which becomes activated by an increased AMP/ATP ratio. During the course of our investigation, Li et al. (2020) reported the generation and characterization of such a deletion mutant of *P. anserina* (in wild-type background “S”) with special emphasis on lignocellulose degradation. In their study, the impact of PaSNF1 on aging and lifespan control was not addressed.

### Ablation of PaSNF1 Affects Development and Increases Lifespan

Deletion of *PaSnf1* was performed following the procedure described in Hamann et al. (2005) in wild-type strain “s” Rizet (1953). The genomic sequence *Pa_2_770* annotated to encode PaSNF1 (UniProt B2B4C1) was replaced by a phleomycin resistance cassette. A double mutant, lacking *PaSnf1* and *PaClpP*, was selected from the progeny of crosses of the newly isolated *PaSnf1* mutant with the previously isolated Δ*PaClpP* strain (Fischer et al., 2013). The correct genotype of strains was verified by Southern blot analysis (see Supplementary Figure 1A). In addition, western blot analysis using a commercially available anti-phospho-α-AMPK antibody failed to detect phosphorylated and thus activated AMPK in the *PaSnf1* deletion strain (Supplementary Figure 1B). As control, a plasmid encoding FLAG::PaSNF1 was introduced in the Δ*PaSnf1* strain. The resulting Δ*PaSnf1*/*Flag::PaSnf1* strain was verified by Southern blot analysis (Supplementary Figure 1C), and production of the FLAG-tagged PaSNF1 protein was demonstrated by western blot analysis (Supplementary Figure 1D).

**FIGURE 2:**
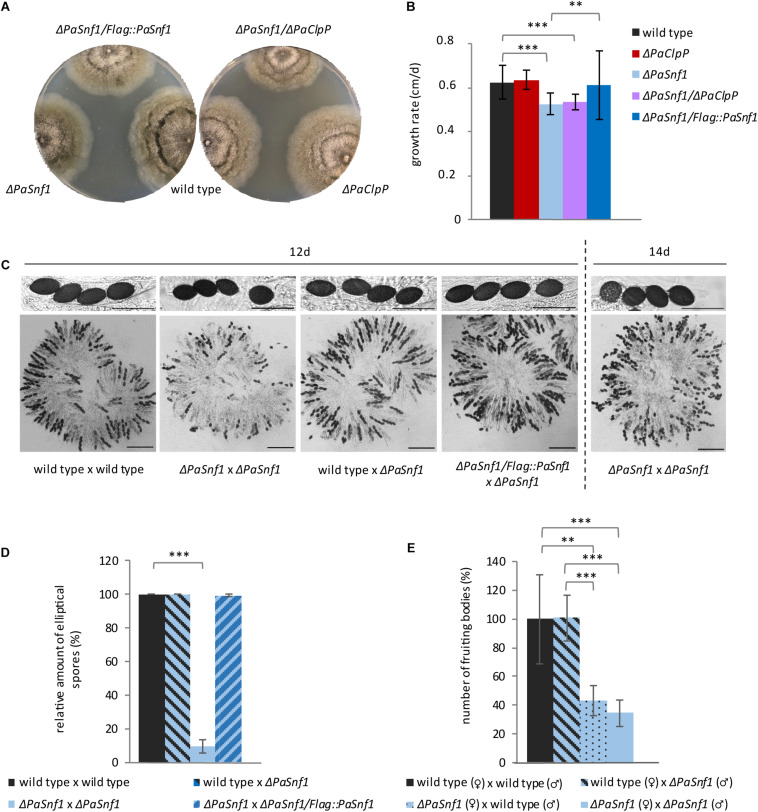
Ablation of PaSNF1 affects growth rate and fertility. **(A)** Phenotypes of Δ*PaSnf1*, Δ*PaClpP*, and Δ*PaSnf1*/Δ*PaClpP*; the complementation strain Δ*PaSnf1*/*Flag::PaSnf1*; and the wild type at the age of 9 days on M2 medium, cultivated at 27°C and in constant light. **(B)** Growth rate of Δ*PaSnf1* (*n* = 28), Δ*PaClpP* (*n* = 22), Δ*PaSnf1*/Δ*PaClpP* (*n* = 25), Δ*PaSnf1*/*Flag::PaSnf1* (*n* = 21), and wild type (*n* = 32) on M2 medium at 27°C and in constant light. **(C)** Content of fruiting bodies from cultures after a 12-day incubation of pairs of opposite mating types (confrontational crosses) of two wild-type strains, of two Δ*PaSnf1* strains, of wild type × Δ*PaSnf1*, or of Δ*PaSnf1*/*Flag::PaSnf1* × Δ*PaSnf1*, respectively. (**C**, right) Content of a fruiting body of the cross of two Δ*PaSnf1* after two additional days of incubation (14 day). Scale bar of fruiting body content corresponds to 250 μm and the scale bar of ascus pictures corresponds to 100 μm. **(D)** Quantification of spore morphology. Only mature black spores (10 fruiting bodies of each cross) were evaluated and the relative amount of elliptical spores is indicated. Evaluated ascospores of specific crosses: wild type × wild type *n* = 1691 spores, Δ*PaSnf1* × Δ*PaSnf1: n* = 760; wild type × Δ*PaSnf1*: *n* = 1595; Δ*PaSnf1*/*Flag::PaSnf1* × Δ*PaSnf1*: *n* = 1423. **(E)** Relative amount of fruiting bodies resulting from the spermatization of the wild type (*n* = 6) and Δ*PaSnf1* (*n* = 6) with different male or female partners. **(B,D,E)** The values presented are mean values ± SD (^∗∗∗^*p* < 0.001; ^∗∗^*p* < 0.01, two-tailed Student’s *t*-test).

Except for a significantly reduced growth rate, the *PaSnf1* deletion strain does not show any obvious macroscopic differences (i.e., pigmentation, aerial hyphae formation) compared to the wild type grown on M2 standard medium (Figures 2A,B). As described earlier, the Δ*PaClpP* strain has a wild-type-like growth rate (Fischer et al., 2013). In the double mutant Δ*PaSnf1*/Δ*PaClpP*, growth rate is reduced like in Δ*PaSnf1* (Figure 2B), suggesting that growth impairment of Δ*PaClpP* is epistatic. Introduction of the *Flag*-tagged *PaSnf1* copy into the *PaSnf1* deletion strain restored the wild-type growth rate (Figures 2A,B). In addition to the reduction in growth rate, the analysis of sexual reproduction revealed severe impairments in the Δ*PaSnf1* strain. In our analyses, we investigated the fertility of *PaSnf1* deletion strains originating from monokaryotic ascospores. These cultures were either of mating type “plus (+)” or “minus (−)” and form fruiting bodies only after contact with a partner of the opposite mating type. At note, mating of *P. anserina* is possible by placing mycelial pieces of the two strains of opposite mating type on opposite positions on one agar plate. After the partners contact each other, the male gametes (spermatia) of each of the two strains fertilize the female gametangia (protoperithecia) and fruiting bodies (perithecia) are subsequently formed. This kind of “confrontational cross” allows to investigate the efficiency and morphological differences in the formation of reproductive structures (perithecia, ascospores) but not the discrimination of the functionality of male gametes and female gametangia, respectively. Such discrimination is possible by spermatization of a growing culture of one mating type (the “acceptor” strain) with spermatia of the opposite mating type isolated from the “donor” strain grown on a separate agar plate. In this case, fruiting bodies are only formed when spermatia are poured on the “acceptor” strain and female and male fertility can be clearly attributed and measured.

In our study, we first investigated the content of the fruiting bodies derived from confrontational crosses of different strains by light microscopy (Figure 2C). In crosses of two wild-type strains of the opposite mating type, normal mature spores occur after 12 days of incubation at 27°C (constant light). In contrast, after this time, only few mature spores are visible in fruiting bodies of Δ*PaSnf1* × Δ*PaSnf1* crosses. However, after two additional days of incubation, in the mutant’s fruiting bodies more mature spores occur (Figure 2C, right) demonstrating that ablation of PaSNF1 delays spore development. Even more, the morphology of the ascospores in these crosses is clearly distinct from those in which one parent is wild type. The ascospores derived from a Δ*PaSnf1* × Δ*PaSnf1* cross are rather round instead of elliptical, suggesting that PaSNF1 is involved in spore morphology regulation. The presence of one *PaSnf1* gene within the fruiting bodies is sufficient (e.g., in a wild type × Δ*PaSnf1* cross) to induce normal spore morphology (Figures 2C,D). Complementation with an ectopically integrated *PaSnf1* gene fused to a *Flag* tag restores spore morphology (Figures 2C,D).

Next, we performed spermatization experiments and found that female fertility is strongly affected in *PaSnf1* deletion strains. While spermatia of the Δ*PaSnf1* strain are readily able to induce fruiting body formation on wild-type acceptor strains, the spermatization of Δ*PaSnf1* by spermatia of the wild type (reciprocal cross) leads to a reduction in fruiting body formation by 57% (Figure 2E). In crosses of male and female Δ*PaSnf1*, the number of fruiting bodies is not further reduced, indicating that it is female fertility that is impaired in the mutant.

**FIGURE 3:**
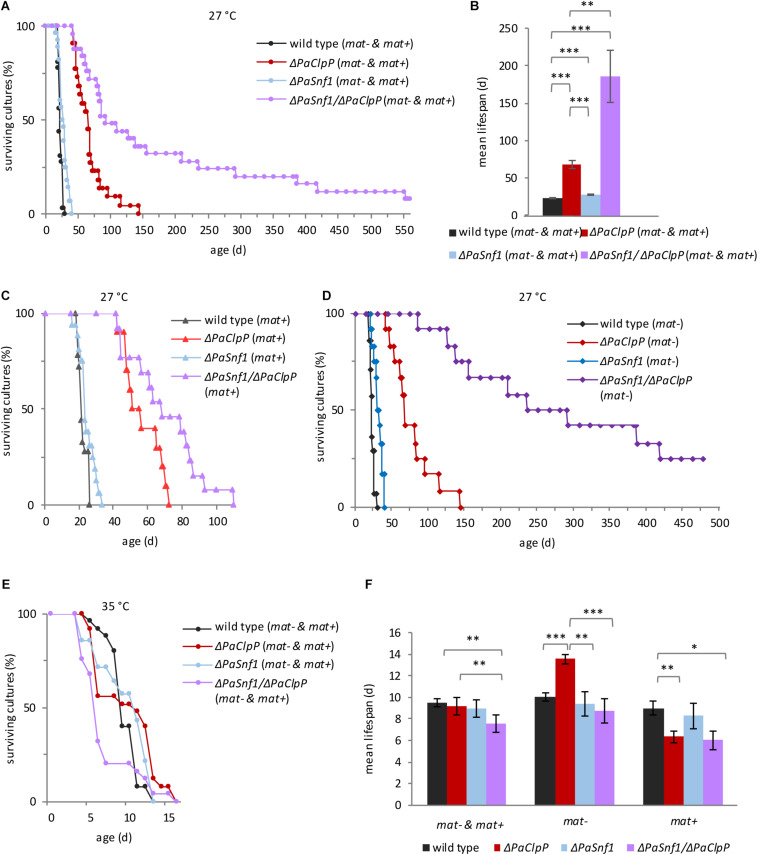
PaSNF1 impacts on lifespan. **(A,B)** Lifespan analysis at 27°C of Δ*PaSnf1* (*n* = 28, *p* < 0.001), Δ*PaClpP* (*n* = 22, *p* < 0.001), Δ*PaSnf1*/Δ*PaClpP* (*n* = 25, *p* < 0.001), and the wild type (*n* = 32), grown on M2 medium in constant light. **(B)** Mean lifespan of cultures from panel **(A)**. **(C)** Lifespan analysis at 27°C of Δ*PaSnf1* (*mat+*) (*n* = 16, *p* = 0.0389), Δ*PaClpP* (*mat+*) (*n* = 10, *p* = 0.0058), Δ*PaSnf1*/Δ*PaClpP* (*mat+*) (*n* = 13, *p* < 0.001), and wild type (*mat+*) (*n* = 18) on M2 medium in constant light. “(*mat+*)” represents the mating type “plus” (*rmp1-2*). **(D)** Lifespan analysis at 27°C of Δ*PaSnf1* (*mat–*) (*n* = 12, *p* < 0.001), Δ*PaClpP* (*mat–*) (*n* = 12, *p* < 0.001), Δ*PaSnf1*/Δ*PaClpP* (*mat–*) (*n* = 12, *p* < 0.001), and wild type (*mat–*) (*n* = 14) on M2 medium in constant light. “(*mat–*)” represents the mating type “minus” (*rmp1-1*). **(E)** Lifespan analysis at 35°C of Δ*PaSnf1* (*n* = 17, *p* = 0.523), Δ*PaClpP* (*n* = 28, *p* = 0.691), Δ*PaSnf1*/Δ*PaClpP* (*n* = 19, *p* = 0.024), and wild type (*n* = 25), grown on M2 medium in constant light. **(F)** Mean lifespan of cultures from panel **(E)**. (**A,B,E**, and left group in panel **F**): the data of strains of both mating types are combined (“*mat– & mat+*”). **(A,C–E**): *p*-values of the lifespan curves in comparison to wild type were determined by SPSS with three different statistic tests. A compilation of all *p*-values is provided in Supplementary Tables 2, 3. **(B,F)** Shown are mean values ± SEM (^∗∗∗^*p* < 0.001; ^∗∗^*p* < 0.01; *p < 0.05, two-tailed Student’s *t*-test).

Finally, we investigated the impact of *PaSnf1* deletion on lifespan. We analyzed individual cultures derived from monokaryotic ascospores grown at 27°C on the M2 medium containing glucose as a carbon source (Figure 3A and Supplementary Table 2). As described earlier (Fischer et al., 2013), Δ*PaClpP* is long-lived. Compared to the wild type, the *PaSnf1* deletion strain shows a 22% mean lifespan extension (Figure 3B and Supplementary Table 2, mean lifespan 28 days vs. wild-type mean lifespan of 23 days). Complementation of Δ*PaSnf1* with an ectopically integrated *Flag*-tagged *PaSnf1* gene restores wild-type lifespan (Supplementary Figure 1E and Supplementary Table 3). Most surprisingly, in contrast to our initial expectation, the Δ*PaClpP*/Δ*PaSnf1* double mutant did not display a wild-type like lifespan. In fact, the simultaneous deletion of the two genes was found to be synergistic leading to a spectacular extension of the mean lifespan to more than 186 days (Figures 3A,B and Supplementary Table 2). This is an increase of at least 174 and 564%, respectively, in comparison to the *PaClpP* and *PaSnf1* single deletion strains. At note, the exact values could not be calculated yet, since some of the double mutant strains are still alive. It is striking that in the double mutant the mortality rate is strongly reduced in particular after about 50% of the investigated individual cultures died (Figure 3A).

This biphasic progression of the survival curve is rather unusual, and therefore, we examined the lifespan data in more detail. In *P. anserina*, it is known that the mating type has an impact on lifespan. In most cases (e.g., wild type), this impact is rather moderate. However, in combination with some genes it can be stronger (Belcour et al., 1991; Contamine et al., 1996, 2004; Sellem et al., 2005; Adam et al., 2012). In fact, this mating type-linked effect is due to two alleles of the *rmp1* gene that are located in very close proximity to the mating-type alleles and therefore are not separated by intrachromosomal recombination during sexual reproduction and co-inherited with the mating type of the parental strain (Contamine et al., 2004). We therefore decided to split the combined lifespan curves of Figure 3A in which mating type “plus” and mating type “minus” isolates are both presented into two mating type-specific curves (Figures 3C,D). Significantly, we found a strong effect of the mating type on lifespan of Δ*PaClpP*/Δ*PaSnf1*. While the lifespan of mating-type “plus” double mutants are only moderately increased, in mating-type “minus” background lifespan is dramatically increased. Thus, the original biphasic progression of the survival curve is due to the fact that the “plus” strains have a shorter lifespan than the “minus” strains. In contrast, the effect of the mating type on lifespan of the wild type and the *PaSnf1* and *PaClpP* single deletion strains was rather moderate (Figures 3C,D and Supplementary Table 3). Because of this observation, we provide lifespan values for both combined mating types (Supplementary Table 2) and lifespans separated for the two mating types (Supplementary Table 3).

From a previous study, we know that the observed lifespan extension may only be valid under the specific laboratory conditions applied in the corresponding study (Weil et al., 2011). In particular, incubation temperature, which – under natural conditions – strongly varies during the day, may have a clear impact. In order to investigate such an impact, we analyzed cultures derived from monokaryotic spores of all strains incubated at 35°C (Figures 3E,F and Supplementary Tables 2,3). All strains showed a strong reduction in lifespan when grown at this temperature. The mean lifespan of wild type, Δ*PaClpP*, and Δ*PaSnf1* is 10, 9, and 10 days, respectively (Figure 3E and Supplementary Table 2) compared to 23, 68, and 28 days at 27°C (Figure 3B and Supplementary Table 2). In wild type and Δ*PaSnf1*, the lifespan at 35°C is not affected by the mating type. However, interestingly, strains ablated for PaCLPP with *rmp1-2* (mating type “plus”) have a strongly reduced lifespan (Figure 3F). In contrast, the Δ*PaClpP* strain with *rmp1-1* allele (mating type “minus”) has a longer lifespan than wild type “minus” (Figure 3F).

### PaSNF1 Is Required for Autophagy

**FIGURE 4:**
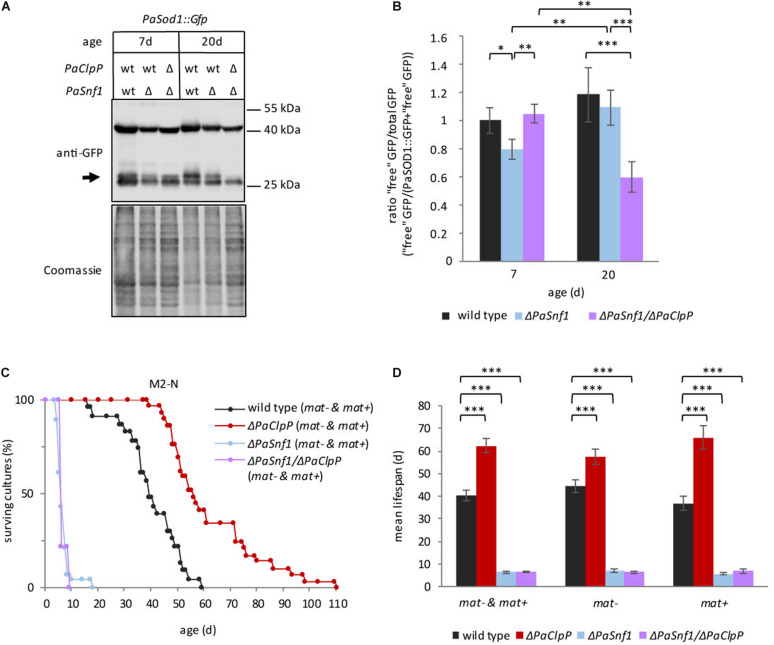
Deletion of *PaSnf1* leads to impaired autophagy. **(A)** Western blot analysis of Δ*PaSnf1* and wild type expressing the autophagy marker gene *PaSod1::Gfp* of 7- (*PaSod1::Gfp n* = 5; Δ*PaSnf1*/*PaSod1::Gfp n* = 4; Δ*PaSnf1*/Δ*PaClpP*/*PaSod1::Gfp n* = 3) and 20-day-old (*PaSod1::Gfp n* = 5; Δ*PaSnf1*/*PaSod1::Gfp n* = 5; Δ*PaSnf1*/Δ*PaClpP*/*PaSod1::Gfp n* = 4) strains. 50 μg of total protein extract was separated in a 12% SDS polyacrylamide gel. After transfer on a PVDF membrane, the gel was stained with Coomassie and serves as loading control. The signal of “free GFP” is marked with an arrow. **(B)** Quantification of the relative amount of “free GFP”/total GFP. The values shown are mean ± SD. **(C)** Lifespan analysis of Δ*PaSnf1* (*n* = 28, *p* < 0.001), Δ*PaClpP* (*n* = 29, *p* < 0.001), Δ*PaSnf1*/Δ*PaClpP* (*n* = 9, *p* < 0.001), and wild type (*n* = 23), grown on M2 medium without nitrogen and with 1.5-fold glucose at 27°C (constant light). *P*-values of the lifespan curves in comparison to wild type were determined by SPSS with three different statistic tests. All *p*-values are provided in Supplementary Tables 2, 3. **(D)** Mean lifespan of cultures from (C) ± SEM. “*mat– & mat+*” represents the mean lifespan taking together both mating types; “*mat–*” shows the mean lifespan of cultures with mating type “minus” (*rmp1-1*). “*mat+*” shows the mean lifespan of cultures with mating type “plus” (*rmp1-2*). (^∗∗∗^: *p* < 0.001; **: *p* < 0.01; *: *p* < 0.05, two-tailed Student’s *t*-test).

AMP-activated protein kinase is known to activate autophagy through different mechanisms, e.g., directly by phosphorylation of ULK1 and BECLIN1 (the mammalian homologs of ATG1 and ATG6, Kim et al., 2011, 2013) and indirectly by inhibiting TOR (Russell et al., 2014). Therefore, we expected that ablation of PaSNF1 results in impaired autophagy. To test this possibility, we measured autophagy in the wild type, Δ*PaSnf1*, and Δ*PaSnf1*/Δ*PaClpP* using the previously established reporter degradation assay (Knuppertz et al., 2014). In this assay, after autophagic degradation of the cytosolic reporter protein PaSOD1::GFP, the GFP moiety remains stable in the fungal vacuole and can be detected by western blot analyses. The results obtained with this reporter are independent of the mating type, and therefore, we presented the data of “plus” and “minus” isolates together. As expected, in 7-day-old Δ*PaSnf1* strains autophagy is significantly impaired and reduced amounts of free GFP are found (Figures 4A,B). At 20 days of age, this difference is reduced and no longer significant, suggesting that aging induces PaSNF1-independent autophagy. Surprisingly, in 7-day-old Δ*PaSnf1*/Δ*PaClpP* autophagy is indistinguishable from that of 7-day-old wild type. In contrast, in 20-day-old cultures autophagic flux is significantly lower in the double mutant compared to wild type. Thus, a beneficial induction of autophagy, as it is known for the single *PaClpP* deletion mutant (Knuppertz and Osiewacz, 2017), is not responsible for the lifespan extension of the double mutant.

Next, we tested whether or not the observed impairment in autophagy of the *PaSnf1* deletion strain affects the ability to respond to nitrogen limitation, a well-known condition inducing autophagy in different organisms including *P. anserina* (Todde et al., 2009; Mukaiyama et al., 2010; Knuppertz et al., 2014; Li et al., 2015). We determined the lifespan of all investigated strains on M2 medium without urea as nitrogen source and supplemented with additional glucose (Figures 4C,D). Nitrogen limitation leads to a pronounced lifespan extension in the wild type (40 days, Figure 4D, vs. 23 days on M2, Figure 3B, respectively, Supplementary Table 2), which is independent of the mating type (Figure 4D and Supplementary Table 3). The mean lifespan of Δ*PaClpP* is slightly reduced compared to the lifespan on M2 media with nitrogen (62 days, Figure 4D, vs. 68 days on M2, Figure 3B, Supplementary Table 2, respectively) but still longer than that of the wild type. In contrast, under nitrogen-replete conditions both Δ*PaSnf1* and Δ*PaSnf1*/Δ*PaClpP* are characterized by a strongly reduced lifespan, which is 7 days for both strains (Figure 4D and Supplementary Table 2) compared to 28 days and at least 186 days on M2 (Figure 3B and Supplementary Table 2). These results identified a pivotal role of PaSNF1 in lifespan control under nitrogen-limiting conditions. Moreover, under these conditions, the deletion of *PaSnf1* is epistatic over the deletion of *PaClpP* again indicating a link of *PaClpP* and *PaSnf1* controlled pathways.

### Ablation of PaSNF1 Affects Mitochondrial Dynamics and Respiration

**FIGURE 5:**
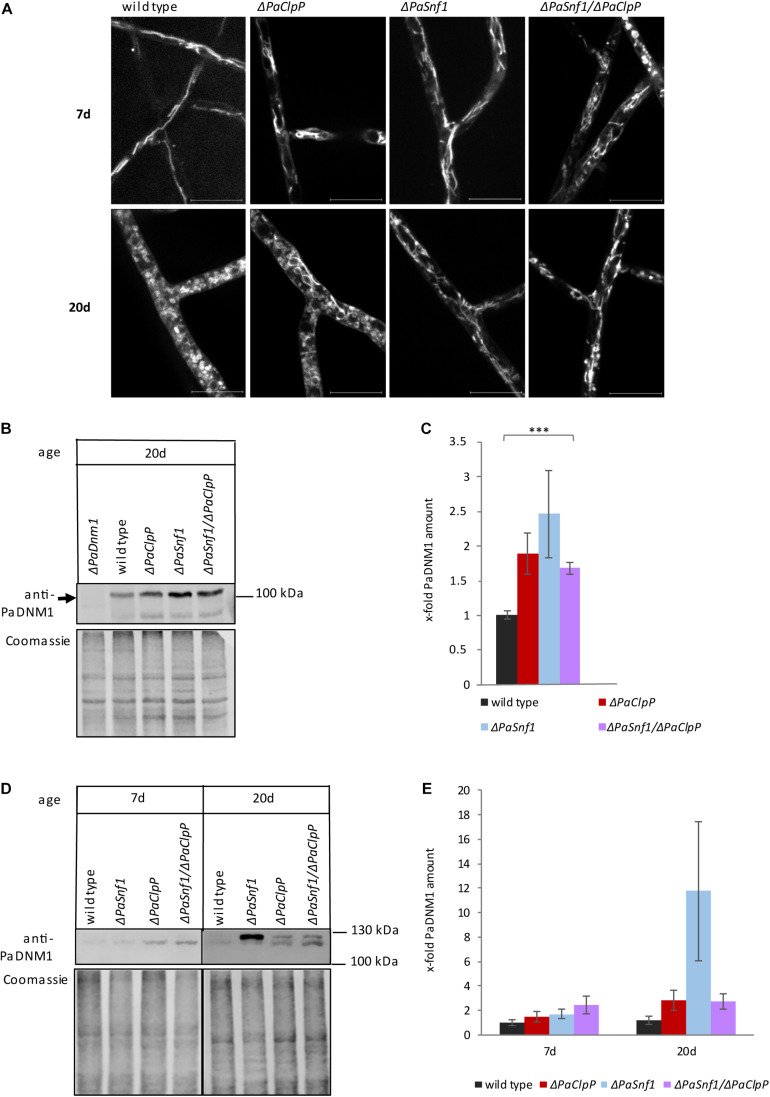
Ablation of PaSNF1 affects mitochondrial morphology. **(A)** Confocal laser scanning fluorescence microscopy with stained mitochondria of 7- and 20-day-old Δ*PaSnf1*, Δ*PaClpP*, Δ*PaSnf1*/Δ*PaClpP*, and the wild type. After aging on M2 medium at 27°C (constant light), pieces of mycelium were used to inoculate microscope slides with a central depression filled with M2 media. After a 1-day incubation at 27°C (constant light), the mycelium was incubated with MitoTracker Red (2 μM, 15 min, in the dark) to visualize mitochondria. The scale bar refers to 10 μm. **(B)** Western blot analysis of 100 μg total protein extracts of 20-day-old Δ*PaSnf1* (*n* = 3), Δ*PaClpP* (*n* = 4), Δ*PaSnf1*/Δ*PaClpP* (*n* = 4), and wild-type strains (*n* = 3) with an anti-PaDNM1 antibody. **(C)** Quantification of PaDNM1 **(B)** after normalization to the Coomassie-stained gel. **(D)** Western blot analysis of 50 μg mitochondrial protein extracts of 7- and 20-day-old Δ*PaSnf1* (*n* = 3), Δ*PaClpP* (*n* = 3), Δ*PaSnf1*/Δ*PaClpP* (*n* = 3), and wild type strains (*n* = 3) with an anti-PaDNM1 antibody. **(E)** Quantification of PaDNM1 **(D)** after normalization to the Coomassie-stained gel. **(B,D)**: 8% SDS polyacrylamide gels were used. After transfer, the gels were stained with Coomassie and served as loading control. The values shown are mean ± SEM (^∗∗∗^*p* < 0.001, two-tailed Student’s *t*-test).

Since AMPK is known to be involved in the regulation mitochondrial homeostasis via mitochondrial biogenesis and dynamics (reviewed in Herzig and Shaw, 2018), we next investigated the impact of the deletion of *PaSnf1* on mitochondria. To this end, we analyzed the mitochondrial morphology of wild type, the two single deletion mutants, and the Δ*PaSnf1*/Δ*PaClpP* double mutant by fluorescence microscopy (Figure 5A). As described earlier, during aging of *P. anserina* mitochondria become fragmented (Scheckhuber et al., 2007). In 20-day-old wild-type hyphae all mitochondria are fragmented, while in Δ*PaClpP* some mitochondria remain filamentous (Figure 5A). Interestingly, we found that both Δ*PaSnf1* and Δ*PaSnf1*/Δ*PaClpP* still contain a substantial fraction of filamentous mitochondria even at 20 days of age – independent of the mating type. Thus, as described in other systems (Toyama et al., 2016; Willis et al., 2018; Chen et al., 2019; Wu et al., 2020), PaSNF1 appears to be crucial for fission of *P. anserina* mitochondria.

Recently, in HeLa cells the translocation of AMPK from the cytosol to mitochondria was demonstrated upon energy stress, thereby regulating mitochondrial division and autophagy (Hu et al., 2020). We wondered whether ablation of PaSNF1 might impair the recruitment of the mitochondrial fission factor PaDNM1 to mitochondria leading to impaired mitochondrial fission. Scheckhuber et al. (2007) showed that the *PaDnm1* gene is only poorly expressed in young cultures. Therefore, we first analyzed the amount of PaDNM1 in total protein extracts of 20-day-old cultures (Figures 5B,C). Interestingly, in all mutants, the amount of PaDNM1 in total protein extracts is higher than in the wild type. However, only the increase in the double mutant is significant (Figure 5C). Subsequently, we investigated whether recruitment of PaDNM1 to mitochondria is altered in the mutants in comparison to the wild type. Surprisingly, on first glance, in purified mitochondria of 20-day-old Δ*PaSnf1* the amount of PaDNM1 appears to be elevated (Figures 5D,E). However, the different biological replicates show a high variability which is not dependent on the mating type. The differences in the abundance of PaDNM1 are not significant, and the ablation of PaSNF1 thus does not impair PaDNM1 recruitment to the mitochondrial fraction. Nonetheless, PaDNM1 function appears to be affected in the mutant strains. While in all young cultures and in 20-day-old wild types only one PaDNM1 protein of about 120 kDa is visible, in 20-day-old mutant strains an additional band of about 125 kDa can be detected (Figure 5D). This band is very prominent in Δ*PaSnf1*, suggesting that PaSNF1 ablation may affect post-translational modification of PaDNM1. It thus is possible that this modification may lead to inactivation of PaDNM1 and, as a consequence, to reduced mitochondrial fission and to filamentous mitochondria even in 20-day-old cultures. In the double mutant as well as in Δ*PaClpP*, both PaDNM1 bands are present in equal amounts. Thus, the observed differences in mitochondrial morphology between these two strains cannot be explained by distinct post-translational modification of PaDNM1.

In search of a possible explanation for the observed differences in mitochondrial morphology, we carefully inspected the transcriptome data of the *PaSnf1* deletion mutant which was recently published by Li et al. (2020). Interestingly, one gene, *PODANS_1_1780*, was found to be strongly upregulated in Δ*PaSnf1* compared to the wild type. The corresponding protein, PODANS_1_1780 (hereafter termed PaMDM38), is a homolog of yeast MDM38 and human LETM1 protein (Nowikovsky et al., 2007; Nakamura et al., 2020), proteins which are implicated in the regulation of mitochondrial morphology. Therefore, we investigated *PaMdm38* transcription by qRT-PCR and found that the gene is indeed upregulated in Δ*PaSnf1* (Supplementary Figure 2), but not in other mutants, suggesting that post-translational modification of PaDNM1 and *PaMdm38* expression is linked.

**FIGURE 6:**
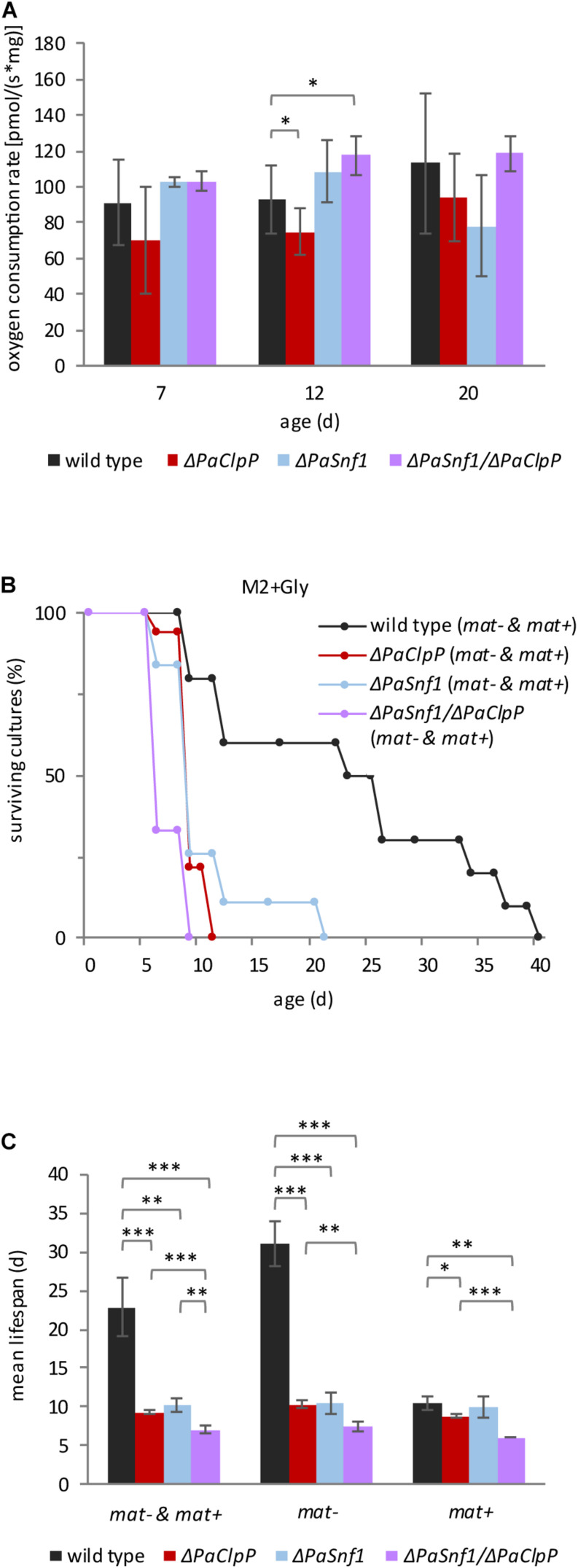
Lack of PaSNF1 does not significantly affect oxygen consumption and impairs growth on glycerol. **(A)** Oxygen consumption rate (OCR) of mycelium from 7-, 12-, and 20-day-old Δ*PaSnf1*, Δ*PaClpP*, Δ*PaSnf1*/Δ*PaClpP*, and wild type. [7 days: Δ*PaSnf1* (*n* = 3), Δ*PaClpP* (*n* = 6), Δ*PaSnf1*/Δ*PaClpP* (*n* = 4), and wild type (*n* = 12) // 12 days: Δ*PaSnf1* (*n* = 4), Δ*PaClpP* (*n* = 6), Δ*PaSnf1*/Δ*PaClpP* (*n* = 4), and wild type (*n* = 13) // 20 days: Δ*PaSnf1* (*n* = 3), Δ*PaClpP* (*n* = 6), Δ*PaSnf1*/Δ*PaClpP* (*n* = 3), and wild type (*n* = 10)]. The values presented are mean ± SD. **(B)** Lifespan analysis of Δ*PaSnf1* (*n* = 19, *p* < 0.001), Δ*PaClpP* (*n* = 18, *p* < 0.001), Δ*PaSnf1*/Δ*PaClpP* (*n* = 9, *p* < 0.001), and wild type (*n* = 10), grown on M2 medium at 27°C with glycerol as carbon source instead of glucose. The *p*-values of the lifespan curves in comparison to wild type were determined by SPSS with three different statistic tests. A compilation of all *p*-values can be found in Supplementary Tables 2, 3. **(C)** Mean lifespan of cultures from panel **(B)**, the values presented are mean ± SEM. “*mat– & mat+*” represent the combined mean lifespan of both mating types; “*mat–*” shows the mean lifespan of cultures with mating type “minus” (*rmp1-1*). “*mat+*” shows the mean lifespan of cultures with mating type “plus” (*rmp1-2*) (^∗∗∗^*p* < 0.001; ^∗∗^*p* < 0.01; ^∗^*p* < 0.05, two-tailed Student’s *t*-test).

Our results indicate that ablation of PaCLPP is able to counteract changes in PaDNM1 recruitment and post-translational modification as well as the induction of *PaMdm38* expression, suggesting that *PaSnf1* affects mitochondrial pathways. We therefore tested mitochondrial function in the different strains by measuring oxygen consumption of fungal mycelium at three different age stages (7, 12, and 20 days) in fresh liquid medium containing 1% glucose (Figure 6A). We found that aging does not significantly affect the oxygen consumption rate (OCR) in the investigated strains. Markedly, the OCR of Δ*PaClpP* is lower than the OCR of the wild type as described before (Knuppertz and Osiewacz, 2017). In contrast, at these conditions, OCR is not significantly affected in Δ*PaSnf1* (Figure 6A). A small but significant increase in OCR is observed in 12-day-old Δ*PaSnf1*/Δ*PaClpP* (Figure 6A). This moderate impact on OCR seen upon PaSNF1 ablation is in agreement with the observation that yeast SNF1 only becomes activated upon glucose depletion (Vallejo et al., 2020). If this response is also true in *P. anserina*, ablation of PaSNF1 should not affect OCR at high glucose concentrations. However, it can be expected that on medium containing non-fermentable glycerol as the sole carbon source, PaSNF1 is important. To test this possibility, we performed a lifespan analysis on M2 medium containing glycerol. On this substrate, all strains show a reduction in mean lifespan. It is 23 days in wild type, 9 days in Δ*PaClpP*, 10 days in Δ*PaSnf1*, and 7 days in Δ*PaSnf1*/Δ*PaClpP*, compared to 23, 68, 28, and at least 186 days on M2 with 1% glucose (Figures 6B,C vs. Figure 3A,B and Supplementary Table 2). We also tested whether or not *rmp1* affects survival on glycerol medium (Supplementary Table 3). Indeed, similar to the situation at 27 and 35°C with 1% glucose, also growth on glycerol is affected by the *rmp1* allele (Figure 6C and Supplementary Table 3). Most intriguingly, the lifespan of wild-type strains is clearly higher in the presence of *rmp1-1* (mating type “minus”) than *rmp1-2* (mating type “plus”). On this medium, *rmp1-1* or *rmp1-2* has no effect in the mutant strains Δ*PaClpP*, Δ*PaSnf1*, and Δ*PaSnf1*/Δ*PaClpP*. Thus, *rmp1-1* ensures the ability to use the non-fermentable carbon source glycerol in the wild type. This ability is reduced in all deletion mutants, clearly demonstrating that they are characterized by impaired mitochondrial function although mitochondria morphology is filamentous.

From these observations, several conclusions can be drawn: First, the lifespan of *P. anserina* carrying the *rmp1-2* allele is reduced on the medium, which requires functional mitochondria indicating a critical lifespan-limiting role of the *rmp1* gene. Second, survival of Δ*PaClpP* is significantly more strongly compromised on glycerol than survival of the wild type. This demonstrates that mitochondrial function is impaired in this mutant, which is in accordance with the observed reduction in OCR. Thus, ablation of PaCLPP affects mitochondrial function independent of the available carbon source. Third, although ablation of PaSNF1 alone does not reduce OCR at 1% glucose, it strongly impairs survival on glycerol containing M2 (Figures 6B,C). Thus, PaSNF1 seems to control mitochondrial function only in the glycerol medium, which suggests that PaSNF1 is required for the metabolic switch from glycolysis to respiration. Obviously, PaCLPP and PaSNF1 affect mitochondrial function independently. Overall, our results uncover a hitherto unknown mechanism by which PaSNF1, RMP1, and PaCLPP synergistically interact with each other and affect the lifespan of *P. anserina*.

## Discussion

The previously generated *P. anserina* mutant in which the gene coding for mitochondrial CLPXP complex as part of the mitochondrial protein quality control network is deleted displays an unexpected pronounced lifespan extension (Fischer et al., 2013). In this mutant, clear changes in respiration were observed (Knuppertz and Osiewacz, 2017). Specifically, we found a reduction in complex I- and II-dependent respiration and the induction of an alternative salicyl hydroxamate-sensitive oxidase (AOX), an enzyme that has repeatedly been demonstrated to be induced in a number of different longevity mutants of *P. anserina* (Scheckhuber and Osiewacz, 2008). Despite that respiration via the alternative pathway is less efficient than that via the standard cytochrome c oxidase (COX)-dependent pathway, we found no significant reduction of the ATP content in the mutant. The observed induction of autophagy in the mutant appears to be responsible for the conservation of cellular ATP.

In the current study, we extended our investigations and first performed a comparative, age-related metabolome analysis of *P. anserina* wild-type and Δ*PaClpP* cultures. This analysis revealed changes of various metabolites. Most significantly, compared to young wild types, the metabolites of the TCA cycle, glycolysis, and amino acid level are affected. In addition, the level of AMP is increased, and ATP level is decreased in young Δ*PaClpP*. On the one hand, this increase in the AMP/ATP ratio suggests that protein kinase SNF1 is activated in the young mutant. However, on the other hand, the amount of 1,6-P hexose is increased in the young mutant compared to wild-type, which might impair SNF1 activation, as it has recently been shown in mammals (Zhang et al., 2017). Thus, based on the metabolome data, the role of SNF1 in Δ*PaClpP* is unclear. To address this issue, we generated a *PaSnf1* deletion mutant in which the catalytic α-subunit of AMPK is ablated and used this mutant to select a Δ*PaClpP*/Δ*PaSnf1* double mutant.

Since SNF1/AMPK is implicated in autophagy induction (Herzig and Shaw, 2018; Tamargo-Gomez and Marino, 2018), we were not surprised to see that ablation of PaSNF1 reduces the autophagic degradation of reporter proteins. Concordantly, the lifespan-extending effect of nitrogen depletion which is visible in wild-type and Δ*PaClpP* strains is lost upon *PaSnf1* deletion. Thus, autophagy induction and the lifespan-promoting role of nitrogen depletion both depend at least partially on PaSNF1.

In our study, we observed a prominent role of PaSNF1 in development. In concordance with a parallel study by Li et al. (2020), we found that the growth rate of cultures is reduced in the *PaSnf1* deletion strain and sexual development is affected. However, in contrast to their study, although delayed in comparison to the wild type, we observed the formation of mature ascospores which are functional and able to form colonies. Moreover, we found that spermatization of cultures derived from a mononuclear ascospore of one mating type with spermatia isolated from a culture of a mononuclear ascospore of the opposite mating type leads to the formation of round spores instead of elliptical spores as they are formed in crosses in which one or both parents are wild type. Clearly, *PaSNF*1 is involved in the control of ascospore formation and morphology. Moreover, we found that it is the formation of female gametangia that is affected in the *PaSnf1* deletion strain. The discrepancies in the two studies are probably due to different designs of the experiments. In their study, Li et al. (2020) used *P. anserina* wild-type strain “S” and we used “s.” Moreover, we used cultures derived from mononucleate small ascospores instead of large binucleate ascospores that, without contact to a second parent, give rise to fruiting body formation. Apart from the investigation of sexual development, Li et al. (2020) investigated the impact of PaSNF1 on sterigmatocystin biosynthesis, lignocellulosic degradation, and stress resistance but did not address the role in aging and lifespan control, the focus of our study.

We found that deletion of *PaSnf1* leads to moderate lifespan extension. More surprisingly, the Δ*PaClpP*/Δ*PaSnf1* double mutant was not characterized by a reversion of the long-lived phenotype of the *PaClpP* deletion mutant as we expected it since the AMP/ATP ratio in this mutant was increased and PaSNF1 is involved in the induction of autophagy and longevity in Δ*PaClpP*. It thus appears that an AMPK-independent induction of autophagy occurs in the *PaClpP* deletion mutant and the PaCLPXP- and PaSNF1-controlled pathways act synergistically. Moreover, it is striking that, in comparison to the wild type and the two single deletion mutants, the maximum lifespan of the double mutant is extremely increased (more than 708%, compared to the wild type) due to a strong decrease of the rate of mortality especially in cultures of mating type “minus.” This dependency on the mating type can be attributed to the *rmp1* gene (*PODANS _1_20180*), a gene that is present in two different alleles in *P. anserina* wild-type strains “s” and “S” and tightly linked to the mating-type locus. In mating type “minus,” the *rmp1-1* allele is found, in mating type “plus” *rmp1-2*. The *rmp1-1* allele is assumed to represent the fully functional version of *rmp1* (Contamine et al., 2004), which encodes an essential 1000 amino acids protein (UniProt Q70GH5). The protein bears a mitochondrial targeting peptide, and a GFP-tagged version of the protein has been localized to filamentous cellular structures and to the cytosol depending on the cell type and developmental stage (Contamine et al., 2004). Moreover, RMP1 has previously been linked to respiratory complex assembly (Sellem et al., 2005). A BLAST analysis identified sequence homologs of this protein only in multicellular ascomycetes. The protein contains a so-called SLS domain which is part of the *Saccharomyces cerevisiae* ScSLS1 protein (UniProt P42900), a mitochondrial integral membrane protein whose ablation does not affect growth on glucose but is lethal on a non-fermentable medium (Rouillard et al., 1996). More recently, this protein has been implicated in mitochondrial gene expression (Bryan et al., 2002). ScSLS1 and the mitochondrial matrix protein ScNAM1 together with mtRNA polymerase have been shown to coordinate transcription and translation of mtDNA-encoded gene products ensuring efficient mitochondrial translation (Rodeheffer and Shadel, 2003).

The prominent role of *rmp1* in wild-type lifespan control is specifically seen on the medium containing glycerol. In the presence of *rmp1-2*, the lifespan of wild type is strongly reduced compared to the wild type that contains the *rmp1-1* allele, suggesting that mitochondrial function strongly depends on the presence of *rmp1-1*. Ablation of PaCLPP, PaSNF1, or both, strongly reduces lifespan on glycerol medium independent of *rmp1*. Thus, ablation of PaCLPP as well as PaSNF1 impairs the ability to use the non-fermentable carbon source glycerol. Interestingly, on first glance, the impaired growth on glycerol does not fit to the morphology of the mitochondria of the *PaSnf1* deletion strains which are filamentous and therefore expected to be functional. However, since AMPK/SNF1 is known to be crucial for mitochondrial fission (Toyama et al., 2016; Willis et al., 2018; Chen et al., 2019; Wu et al., 2020), filamentous mitochondria, which are observed even at late age in the *PaSnf1* deletion mutant, rather seem to reflect the inability to undergo fission than being indicative for functional mitochondria. Supporting this idea, we observed an altered migration behavior of the fission factor PaDNM1 in the *PaSnf1* deletion strains.

In yeast, SNF1 controls glycolytic flux and mitochondrial respiration in a glucose-dependent manner. At 1% glucose, a concentration which we used in M2 medium in our studies with *P. anserina*, ScSNF1 sustains glycolytic flux and increases respiration (Martinez-Ortiz et al., 2019). Such a function of PaSNF1 in the Δ*PaClpP* background appears to be not possible because, as revealed by the metabolome analysis, glycolytic flux seems to be impaired in the *PaClpP* deletion strain leading to the accumulation of C6 sugars and depletion of C3 carbohydrates and because complex I- and complex II-dependent respiration is impaired (Knuppertz and Osiewacz, 2017). We suggest that in this situation functional PaSNF1 is detrimental and deletion of *PaSnf1* has a beneficial effect on a medium with 1% glucose. Interestingly, this synergistic effect is strongly reinforced in the presence of the *rmp1-1* allele, bringing mitochondrial translation efficiency into play. In mice, CLPP ablation was found to decrease mitochondrial translation (Szczepanowska et al., 2016). In wild-type mice, CLPP regulates the turnover of ERAL1, a putative 12S rRNA chaperone, which, for efficient translation, has to be timely removed from maturating mitoribosomes. Accumulation of ERAL1 due to ablation of CLPP delays maturation of mitoribosomes (Szczepanowska et al., 2016), thereby diminishing protein synthesis. In certain settings, this effect on protein biosynthesis may be beneficial. For instance, in a mouse mutant with dysregulated mitochondrial translation, *ClpP* deletion has been found to result in a robust lifespan extension (Seiferling et al., 2016). In the corresponding double mutant, more proficient vs. abortive protein synthesis has been observed in mitochondria. It appears that the delay in mitoribosome maturation in *ClpP* mice allows to overcome erroneous translation and ameliorates the phenotype by increasing the lifespan. It is well documented that not only protein quality control is affected during aging of biological systems but also protein synthesis (Anisimova et al., 2018; Klaips et al., 2018). The latter not only holds true for cytosolic protein synthesis but also is relevant for mitochondrial translation as it has been shown in human skeletal muscle (Rooyackers et al., 1996). In *P. anserina*, inhibition of mitochondrial translation by streptomycin, tiamulin, and other inhibitors results in a robust lifespan extension (Esser and Tudzynski, 1977; Tudzynski and Esser, 1977, 1979), which is not seen upon application of inhibitors for cytosolic translation (Tudzynski and Esser, 1977). Thus, mitochondrial rather than cytosolic translation seems to critically intervene into aging. Ablation of PaCLPP resulting in a slowdown of mitochondrial translation may mitigate the consequences of age-dependent impairments in mitochondrial translation leading to the observed lifespan extension of the *PaClpP* deletion mutant. Since ablation of PaSNF1 impairs the switch from glycolysis to respiration, a functional *rmp1* allele is not required, explaining the rather minor effect of the mating type on lifespan. However, concomitant ablation of PaCLPP strongly challenges mitochondrial function and, under these conditions, a fully functional *rmp1* allele is highly beneficial, leading to an extreme increase in lifespan.

Such a lifespan extension is observed not only under standard growth conditions of 27°C but also under heat stress conditions at 35°C. In mammals, the impact of heat stress on mitochondria has been studied. Wilkening et al. (2018) found mitochondrial translation elongation factor Tu to be highly aggregation-prone even under mild heat stress conditions. Thus, under heat stress conditions, mitochondrial translation becomes reduced. In *P. anserina*, such a reduction in mitochondrial translation may be beneficial in Δ*PaClpP* incubated at 35°C leading to lifespan extension (Figure 3F). However, again, lifespan extension strongly depends on the presence of the *rmp1-1* allele (mating-type “minus”), indicating that functional RMP1 is required for this effect.

Overall, the identification of interactions between the proteolytic pathway controlled by PaCLPP, RMP1, a putative component of the mitochondrial transcription/translation apparatus, and energy metabolism governed by PaSNF1 as the central cellular energy sensor described in the current study calls for further detailed investigations to unravel the precise underlying molecular mechanisms. It will be interesting to see how far these mechanisms are unique for *P. anserina* or conserved among species. From the available data, the view is mixed. Common substrates of complex I of the respiratory chain have been identified in *P. anserina*, mammals, and *A. thaliana*. Other substrates differ among systems. Moreover, the yeasts *S. cerevisiae* and *Schizosaccharomyces pombe* lack CLPP and RMP1, the latter of which appears to be specific for multicellular fungi. Nevertheless, work using different biological systems will be instrumental to unraveling the variety of different biological facets of the molecular pathways and their variations, which have been reported in this study, in aging and lifespan control. Such work will certainly also provide important information concerning the role of CLPXP in human health and disease, which has been found in previous investigation (Gispert et al., 2013; Cole et al., 2015; Seo et al., 2016) and certainly will provide clues for therapeutical interventions.

## Data Availability Statement

The original contributions presented in the study are included in the article/Supplementary Material, further inquiries can be directed to the corresponding author/s.

## Author Contributions

HO initiated and supervised this study. DH, AH, and HO designed the experiments and analyzed the data. DH, EK, and AH performed the experiments. AH and HD wrote the manuscript. All authors contributed to the article and approved the submitted version.

## Conflict of Interest

The authors declare that the research was conducted in the absence of any commercial or financial relationships that could be construed as a potential conflict of interest.

## References

[B1] Adam C. Picard M. Déquard-Chablat M. Sellem C. H. Denmat S. H. Contamine V. (2012). Biological roles of the *Podospora anserina* mitochondrial Lon protease and the importance of its N-domain. *PLoS One* 7:e38138. 10.1371/journal.pone.0038138 22693589 PMC3364969

[B2] Anisimova A. S. Alexandrov A. I. Makarova N. E. Gladyshev V. N. Dmitriev S. E. (2018). Protein synthesis and quality control in aging. *Aging (Albany NY)* 10 4269–4288. 10.18632/aging.101721 30562164 PMC6326689

[B3] Baker M. J. Tatsuta T. Langer T. (2011). Quality control of mitochondrial proteostasis. *Cold Spring Harb. Perspect. Biol.* 3:a007559. 10.1101/cshperspect.a007559 21628427 PMC3119916

[B4] Belcour L. Begel O. Picard-Bennoun M. (1991). A site-specific deletion in mitochondrial DNA of *Podospora* is under the control of nuclear genes. *Proc. Natl. Acad. Sci. U.S.A.* 88 3579–3583. 10.1073/pnas.88.9.3579 2023905 PMC51495

[B5] Borghouts C. Kimpel E. Osiewacz H. D. (1997). Mitochondrial DNA rearrangements of *Podospora anserina* are under the control of the nuclear gene grisea. *Proc. Natl. Acad. Sci. U.S.A.* 94 10768–10773. 10.1073/pnas.94.20.10768 9380708 PMC23480

[B6] Borghouts C. Scheckhuber C. Q. Werner A. Osiewacz H. D. (2002). Respiration, copper availability and SOD activity in *P. anserina* strains with different lifespan. *Biogerontology* 3 143–153. 10.1023/a:1015696404723 12075133

[B7] Borghouts C. Werner A. Elthon T. Osiewacz H. D. (2001). Copper-modulated gene expression and senescence in the filamentous fungus *Podospora anserina*. *Mol. Cell Biol.* 21 390–399. 10.1128/MCB.21.2.390-399.2001 11134328 PMC86578

[B8] Breitenbach M. Laun P. Dickinson J. R. Klocker A. Rinnerthaler M. Dawes I. W. (2012). “The role of mitochondria in the aging processes of yeast,” in *Aging Research in Yeast*, eds Breitenbach M. Jazwinski S. M. Laun P. (Dordrecht: Springer Netherlands), 55–78. 10.1007/978-94-007-2561-4_3 22094417

[B9] Brust D. Hamann A. Osiewacz H. D. (2010). Deletion of *PaAif2* and *PaAmid2*, two genes encoding mitochondrial AIF-like oxidoreductases of *Podospora anserina*, leads to increased stress tolerance and lifespan extension. *Curr. Genet.* 55 225–235. 10.1007/s00294-010-0295-1 20306265

[B10] Bryan A. C. Rodeheffer M. S. Wearn C. M. Shadel G. S. (2002). Sls1p is a membrane-bound regulator of transcription-coupled processes involved in *Saccharomyces cerevisiae* mitochondrial gene expression. *Genetics* 160 75–82. 11805046 10.1093/genetics/160.1.75 PMC1461927

[B11] Chen Z. Lei C. Wang C. Li N. Srivastava M. Tang M. (2019). Global phosphoproteomic analysis reveals ARMC10 as an AMPK substrate that regulates mitochondrial dynamics. *Nat. Commun.* 10:104. 10.1038/s41467-018-08004-0 30631047 PMC6328551

[B12] Cole A. Wang Z. Coyaud E. Voisin V. Gronda M. Jitkova Y. (2015). Inhibition of the mitochondrial protease ClpP as a therapeutic strategy for human acute myeloid leukemia. *Cancer Cell* 27 864–876. 10.1016/j.ccell.2015.05.004 26058080 PMC4461837

[B13] Contamine V. Lecellier G. Belcour L. Picard M. (1996). Premature death in *Podospora anserina*: sporadic accumulation of the deleted mitochondrial genome, translational parameters and innocuity of the mating types. *Genetics* 144 541–555. 8889519 10.1093/genetics/144.2.541 PMC1207549

[B14] Contamine V. Zickler D. Picard M. (2004). The *Podospora rmp1* gene implicated in nucleus-mitochondria cross-talk encodes an essential protein whose subcellular location is developmentally regulated. *Genetics* 166 135–150. 10.1534/genetics.166.1.135 15020413 PMC1470695

[B15] Cortopassi G. A. Arnheim N. (1990). Detection of a specific mitochondrial DNA deletion in tissues of older humans. *Nucleic Acids Res.* 18 6927–6933. 10.1093/nar/18.23.6927 2263455 PMC332752

[B16] Cummings D. J. Belcour L. Grandchamp C. (1979). Mitochondrial DNA from *Podospora anserina* and the occurrence of multimeric circular DNA in senescent cultures. *Mol. Gen. Genet.* 171 239–250. 10.1007/BF00267578 286868

[B17] Esser K. (1974). “Podospora anserina,” in *Handbook of Genetics*, ed. King R. C. (New York, NY: Plenum Press), 531–551.

[B18] Esser K. Tudzynski P. (1977). Prevention of senescence in the ascomycete *Podospora anserina* by the antibiotic tiamulin. *Nature* 265 454–456. 10.1038/265454a0 834297

[B19] Fischer F. Filippis C. Osiewacz H. D. (2015a). RCF1-dependent respiratory supercomplexes are integral for lifespan-maintenance in a fungal ageing model. *Sci. Rep.* 5:12697. 10.1038/srep12697 26220011 PMC4518240

[B20] Fischer F. Hamann A. Osiewacz H. D. (2012). Mitochondrial quality control: an integrated network of pathways. *Trends Biochem. Sci.* 37 284–292. 10.1016/j.tibs.2012.02.004 22410198

[B21] Fischer F. Langer J. D. Osiewacz H. D. (2015b). Identification of potential mitochondrial CLPXP protease interactors and substrates suggests its central role in energy metabolism. *Sci. Rep.* 5:18375. 10.1038/srep18375 26679294 PMC4683621

[B22] Fischer F. Weil A. Hamann A. Osiewacz H. D. (2013). Human CLPP reverts the longevity phenotype of a fungal *ClpP* deletion strain. *Nat. Commun.* 4:1397. 10.1038/ncomms2397 23360988 PMC3562451

[B23] Gispert S. Parganlija D. Klinkenberg M. Dröse S. Wittig I. Mittelbronn M. (2013). Loss of mitochondrial peptidase *Clpp* leads to infertility, hearing loss plus growth retardation via accumulation of CLPX, mtDNA and inflammatory factors. *Hum. Mol. Genet.* 22 4871–4887. 10.1093/hmg/ddt338 23851121 PMC7108587

[B24] Gredilla R. Grief J. Osiewacz H. D. (2006). Mitochondrial free radical generation and lifespan control in the fungal aging model *Podospora anserina*. *Exp. Gerontol.* 41 439–447. 10.1016/j.exger.2006.01.010 16530367

[B25] Hamann A. Krause K. Werner A. Osiewacz H. D. (2005). A two-step protocol for efficient deletion of genes in the filamentous ascomycete *Podospora anserina*. *Curr. Genet.* 48 270–275. 10.1007/s00294-005-0018-1 16160832

[B26] Herzig S. Shaw R. J. (2018). AMPK: guardian of metabolism and mitochondrial homeostasis. *Nat. Rev. Mol. Cell Biol.* 19 121–135. 10.1038/nrm.2017.95 28974774 PMC5780224

[B27] Hu Y. Chen H. Zhang L. Lin X. Li X. Zhuang H. (2020). The AMPK-MFN2 axis regulates MAM dynamics and autophagy induced by energy stresses. *Autophagy* 10.1080/15548627.2020.1749490 [Epub ahead of print]. 32249716 PMC8143230

[B28] Kim J. Kim Y. C. Fang C. Russell R. C. Kim J. H. Fan W. (2013). Differential regulation of distinct Vps34 complexes by AMPK in nutrient stress and autophagy. *Cell* 152 290–303. 10.1016/j.cell.2012.12.016 23332761 PMC3587159

[B29] Kim J. Kundu M. Viollet B. Guan K.-L. (2011). AMPK and mTOR regulate autophagy through direct phosphorylation of Ulk1. *Nat. Cell Biol.* 13 132–141. 10.1038/ncb2152 21258367 PMC3987946

[B30] Klaips C. L. Jayaraj G. G. Hartl F. U. (2018). Pathways of cellular proteostasis in aging and disease. *J. Cell Biol.* 217 51–63. 10.1083/jcb.201709072 29127110 PMC5748993

[B31] Knuppertz L. Hamann A. Pampaloni F. Stelzer E. Osiewacz H. D. (2014). Identification of autophagy as a longevity-assurance mechanism in the aging model *Podospora anserina*. *Autophagy* 10 822–834. 10.4161/auto.28148 24584154 PMC5119060

[B32] Knuppertz L. Osiewacz H. D. (2016). Orchestrating the network of molecular pathways affecting aging: role of nonselective autophagy and mitophagy. *Mech. Ageing Dev.* 153 30–40. 10.1016/j.mad.2016.01.003 26814678

[B33] Knuppertz L. Osiewacz H. D. (2017). Autophagy compensates impaired energy metabolism in CLPXP-deficient *Podospora anserina* strains and extends healthspan. *Aging Cell* 16 704–715. 10.1111/acel.12600 28449241 PMC5506401

[B34] Kück U. Osiewacz H. D. Schmidt U. Kappelhoff B. Schulte E. Stahl U. (1985). The onset of senescence is affected by DNA rearrangements of a discontinuous mitochondrial gene in *Podospora anserina*. *Curr. Genet.* 9 373–382. 10.1007/BF00421608 2836091

[B35] Kück U. Stahl U. Esser K. (1981). Plasmid-like DNA is part of mitochondrial DNA in *Podospora anserina*. *Curr. Genet.* 3 151–156. 10.1007/BF00365719 24190061

[B36] Kunstmann B. Osiewacz H. D. (2008). Over-expression of an S-adenosylmethionine-dependent methyltransferase leads to an extended lifespan of *Podospora anserina* without impairments in vital functions. *Aging Cell* 7 651–662. 10.1111/j.1474-9726.2008.00412.x 18616635

[B37] Kunstmann B. Osiewacz H. D. (2009). The S-adenosylmethionine dependent O-methyltransferase PaMTH1: a longevity assurance factor protecting *Podospora anserina* against oxidative stress. *Aging (Albany NY)* 1 328–334. 10.18632/aging.100029 20157520 PMC2806012

[B38] Lecellier G. Silar P. (1994). Rapid methods for nucleic acids extraction from Petri dish-grown mycelia. *Curr. Genet.* 25 122–123. 10.1007/BF00309536 8087879

[B39] Li D. Song J. Z. Li H. Shan M. H. Liang Y. Zhu J. (2015). Storage lipid synthesis is necessary for autophagy induced by nitrogen starvation. *FEBS Lett.* 589 269–276. 10.1016/j.febslet.2014.11.050 25500271

[B40] Li Y. Yan P. Lu X. Qiu Y. Liang S. Liu G. (2020). Involvement of PaSNF1 in fungal development, tterigmatocystin biosynthesis, and lignocellulosic degradation in the filamentous fungus *Podospora anserina*. *Front. Microbiol.* 11:1038. 10.3389/fmicb.2020.01038 32587577 PMC7299030

[B41] Linnane A. W. Marzuki S. Ozawa T. Tanaka M. (1989). Mitochondrial DNA mutations as an important contributor to ageing and degenerative diseases. *Lancet* 1 642–645. 10.1016/s0140-6736(89)92145-4 2564461

[B42] Luce K. Osiewacz H. D. (2009). Increasing organismal healthspan by enhancing mitochondrial protein quality control. *Nat. Cell Biol.* 11 852–858. 10.1038/ncb1893 19543272

[B43] Luce K. Weil A. C. Osiewacz H. D. (2010). Mitochondrial protein quality control systems in aging and disease. *Adv. Exp. Med. Biol.* 694 108–125. 10.1007/978-1-4419-7002-2_9 20886760

[B44] Marcou D. (1962). Notion de longevite et nature cytoplasmatique du determinant de senescence chez quelques champignons. *Ann. Sci. Nat. Biol. Bot. Veg.* 2, 653–764.

[B45] Martinez-Ortiz C. Carrillo-Garmendia A. Correa-Romero B. F. Canizal-Garcia M. Gonzalez-Hernandez J. C. Regalado-Gonzalez C. (2019). *SNF1* controls the glycolytic flux and mitochondrial respiration. *Yeast* 36 487–494. 10.1002/yea.3399 31074533

[B46] Melov S. Hertz G. Z. Stormo G. D. Johnson T. E. (1994). Detection of deletions in the mitochondrial genome of *Caenorhabditis elegans*. *Nucleic Acids Res.* 22 1075–1078. 10.1093/nar/22.6.1075 8152911 PMC307932

[B47] Mukaiyama H. Nakase M. Nakamura T. Kakinuma Y. Takegawa K. (2010). Autophagy in the fission yeast *Schizosaccharomyces pombe*. *FEBS Lett.* 584 1327–1334. 10.1016/j.febslet.2009.12.037 20036658

[B48] Nakamura S. Matsui A. Akabane S. Tamura Y. Hatano A. Miyano Y. (2020). The mitochondrial inner membrane protein LETM1 modulates cristae organization through its LETM domain. *Commun. Biol.* 3 99. 10.1038/s42003-020-0832-5 PMC7058069 32139798

[B49] Nowikovsky K. Reipert S. Devenish R. J. Schweyen R. J. (2007). Mdm38 protein depletion causes loss of mitochondrial K^+^/H^+^ exchange activity, osmotic swelling and mitophagy. *Cell Death Differ.* 14 1647–1656. 10.1038/sj.cdd.4402167 17541427

[B50] Osiewacz H. D. (1994). A versatile shuttle cosmid vector for the efficient construction of genomic libraries and for the cloning of fungal genes. *Curr. Genet.* 26 87–90. 10.1007/BF00326309 7954902

[B51] Osiewacz H. D. (2002). Aging in fungi: role of mitochondria in *Podospora anserina*. *Mech. Ageing Dev.* 123 755–764. 10.1016/s0047-6374(01)00421-3 11869733

[B52] Osiewacz H. D. (2010). Role of mitochondria in aging and age-related disease. *Exp. Gerontol.* 45:465. 10.1016/j.exger.2010.05.001 20451599

[B53] Osiewacz H. D. Esser K. (1984). The mitochondrial plasmid of *Podospora anserina*: a mobile intron of a mitochondrial gene. *Curr. Genet.* 8 299–305. 10.1007/BF00419728 24177799

[B54] Osiewacz H. D. Hamann A. Zintel S. (2013). Assessing organismal aging in the filamentous fungus *Podospora anserina*. *Methods Mol. Biol.* 965 439–462. 10.1007/978-1-62703-239-1_29 23296676

[B55] Osiewacz H. D. Skaletz A. Esser K. (1991). Integrative transformation of the ascomycete *Podospora anserina*: identification of the mating-type locus on chromosome VII of electrophoretically separated chromosomes. *Appl. Microbiol. Biotechnol.* 35 38–45. 10.1007/BF00180633 1367277

[B56] Petereit J. Duncan O. Murcha M. W. Fenske R. Cincu E. Cahn J. (2020). Mitochondrial CLPP2 assists coordination and homeostasis of respiratory complexes. *Plant Physiol.* 184 148–164. 10.1104/pp.20.00136 32571844 PMC7479914

[B57] Petersen K. F. Befroy D. Dufour S. Dziura J. Ariyan C. Rothman D. L. (2003). Mitochondrial dysfunction in the elderly: possible role in insulin resistance. *Science* 300 1140–1142. 10.1126/science.1082889 12750520 PMC3004429

[B58] Pfaffl M. W. (2001). A new mathematical model for relative quantification in real-time RT-PCR. *Nucleic Acids Res.* 29:e45. 10.1093/nar/29.9.e45 11328886 PMC55695

[B59] Rizet G. (1953). Impossibility of obtaining uninterrupted and unlimited multiplication of the ascomycete *Podospora anserina*. *C. R. Hebd. Seances Acad. Sci.* 237 838–855. 13107134

[B60] Rodeheffer M. S. Shadel G. S. (2003). Multiple interactions involving the amino-terminal domain of yeast mtRNA polymerase determine the efficiency of mitochondrial protein synthesis. *J. Biol. Chem.* 278 18695–18701. 10.1074/jbc.M301399200 12637560 PMC2606056

[B61] Rooyackers O. E. Adey D. B. Ades P. A. Nair K. S. (1996). Effect of age on *in vivo* rates of mitochondrial protein synthesis in human skeletal muscle. *Proc. Natl. Acad. Sci. U.S.A.* 93 15364–15369. 10.1073/pnas.93.26.15364 8986817 PMC26410

[B62] Rouillard J. M. Dufour M. E. Dujardin G. Lacroute F. Theunissen B. Mandart E. (1996). *SLS1*, a new *Saccharomyces cerevisiae* gene involved in mitochondrial metabolism, isolated as a synthetic lethal in association with an *SSM4* deletion. *Mol. Gen. Genet.* 252 700–708. 10.1007/BF02173976 8917313

[B63] Russell R. C. Yuan H.-X. Guan K.-L. (2014). Autophagy regulation by nutrient signaling. *Cell Res.* 24 42–57. 10.1038/cr.2013.166 24343578 PMC3879708

[B64] Sambrook J. Fritsch E. F. Maniatis T. (1989). *Molecular Cloning: A Laboratory Manual.* Cold Spring Harbour: Cold Spring Harbour Press.

[B65] Scheckhuber C. Q. Erjavec N. Tinazli A. Hamann A. Nyström T. Osiewacz H. D. (2007). Reducing mitochondrial fission results in increased life span and fitness of two fungal ageing models. *Nat. Cell Biol.* 9 99–105. 10.1038/ncb1524 17173038

[B66] Scheckhuber C. Q. Osiewacz H. D. (2008). *Podospora anserina*: a model organism to study mechanisms of healthy ageing. *Mol. Genet. Genomics* 280 365–374. 10.1007/s00438-008-0378-6 18797929

[B67] Seiferling D. Szczepanowska K. Becker C. Senft K. Hermans S. Maiti P. (2016). Loss of CLPP alleviates mitochondrial cardiomyopathy without affecting the mammalian UPRmt. *EMBO Rep.* 17 953–964. 10.15252/embr.201642077 27154400 PMC4931557

[B68] Sellem C. H. Lecellier G. Belcour L. (1993). Transposition of a group II intron. *Nature* 366 176–178. 10.1038/366176a0 8232558

[B69] Sellem C. H. Lemaire C. Lorin S. Dujardin G. Sainsard-Chanet A. (2005). Interaction between the *oxa1* and *rmp1* genes modulates respiratory complex assembly and life span in *Podospora anserina*. *Genetics* 169 1379–1389. 10.1534/genetics.104.033837 15545650 PMC1449539

[B70] Seo J. H. Rivadeneira D. B. Caino M. C. Chae Y. C. Speicher D. W. Tang H. Y. (2016). The mitochondrial unfoldase-peptidase complex ClpXP controls bioenergetics stress and metastasis. *PLoS Biol.* 14:e1002507. 10.1371/journal.pbio.1002507 27389535 PMC4936714

[B71] Stahl U. Lemke P. A. Tudzynski P. Kuck U. Esser K. (1978). Evidence for plasmid like DNA in a filamentous fungus, the ascomycete *Podospora anserina*. *Mol. Gen. Genet.* 162 341–343. 10.1007/BF00268860 683172

[B72] Szczepanowska K. Maiti P. Kukat A. Hofsetz E. Nolte H. Senft K. (2016). CLPP coordinates mitoribosomal assembly through the regulation of ERAL1 levels. *EMBO J.* 35 2566–2583. 10.15252/embj.201694253 27797820 PMC5283601

[B73] Tamargo-Gomez I. Marino G. (2018). AMPK: regulation of metabolic dynamics in the context of autophagy. *Int. J. Mol. Sci.* 19:3812. 10.3390/ijms19123812 30501132 PMC6321489

[B74] Todde V. Veenhuis M. van der Klei I. J. (2009). Autophagy: principles and significance in health and disease. *Biochim. Biophys. Acta* 1792 3–13. 10.1016/j.bbadis.2008.10.016 19022377

[B75] Toyama E. Q. Herzig S. Courchet J. Lewis T. L. Losón O. C. Hellberg K. (2016). AMP-activated protein kinase mediates mitochondrial fission in response to energy stress. *Science* 351 275–281. 10.1126/science.aab4138 26816379 PMC4852862

[B76] Tudzynski P. Esser K. (1977). Inhibitors of mitochondrial function prevent senescence in the ascomycete *Podospora anserina*. *Mol. Gen. Genet.* 153 111–113. 10.1007/BF01036003 887069

[B77] Tudzynski P. Esser K. (1979). Chromosomal and extrachromosomal control of senescence in the ascomycete *Podospora anserina*. *Mol. Gen. Genet.* 173 71–84. 10.1007/BF00267692 288965

[B78] Vallejo B. Peltier E. Garrigós V. Matallana E. Marullo P. Aranda A. (2020). Role of *Saccharomyces cerevisiae* nutrient signaling pathways during winemaking: a phenomics approach. *Front. Bioeng. Biotechnol.* 8:853. 10.3389/fbioe.2020.00853 32793580 PMC7387434

[B79] Wallace D. C. (1992). Mitochondrial genetics: a paradigm for aging and degenerative diseases? *Science* 256 628–632. 10.1126/science.1533953 1533953

[B80] Weil A. Luce K. Dröse S. Wittig I. Brandt U. Osiewacz H. D. (2011). Unmasking a temperature-dependent effect of the *P. anserina* i -AAA protease on aging and development. *Cell Cycle* 10 4280–4290. 10.4161/cc.10.24.18560 22134244 PMC3272260

[B81] Wen X. Klionsky D. J. (2016). An overview of macroautophagy in yeast. *J. Mol. Biol.* 428 1681–1699. 10.1016/j.jmb.2016.02.021 26908221 PMC4846508

[B82] Wilkening A. Rüb C. Sylvester M. Voos W. (2018). Analysis of heat-induced protein aggregation in human mitochondria. *J. Biol. Chem.* 293 11537–11552. 10.1074/jbc.RA118.002122 29895621 PMC6065183

[B83] Willis S. D. Stieg D. C. Ong K. L. Shah R. Strich A. K. Grose J. H. (2018). Snf1 cooperates with the CWI MAPK pathway to mediate the degradation of Med13 following oxidative stress. *Microb. Cell* 5 357–370. 10.15698/mic2018.08.641 30175106 PMC6116281

[B84] Wu X. Luo J. Liu H. Cui W. Guo K. Zhao L. (2020). Recombinant adiponectin peptide ameliorates brain injury following intracerebral hemorrhage by suppressing astrocyte-derived inflammation via the inhibition of Drp1-mediated mitochondrial fission. *Transl. Stroke Res.* 11 924–939. 10.1007/s12975-019-00768-x 31902083

[B85] Yin Z. Pascual C. Klionsky D. J. (2016). Autophagy: machinery and regulation. *Microb. Cell* 3 588–596. 10.15698/mic2016.12.546 28357331 PMC5348978

[B86] Zhang C.-S. Hawley S. A. Zong Y. Li M. Wang Z. Gray A. (2017). Fructose-1,6-bisphosphate and aldolase mediate glucose sensing by AMPK. *Nature* 548 112–116. 10.1038/nature23275 28723898 PMC5544942

[B87] Zintel S. Schwitalla D. Luce K. Hamann A. Osiewacz H. D. (2010). Increasing mitochondrial superoxide dismutase abundance leads to impairments in protein quality control and ROS scavenging systems and to lifespan shortening. *Exp. Gerontol.* 45 525–532. 10.1016/j.exger.2010.01.006 20080171

