# Supplementary material for: Simultaneous Ablation of the Catalytic AMPK α-Subunit SNF1 and Mitochondrial Matrix Protease CLPP Results in Pronounced Lifespan Extension
Source: Front Cell Dev Biol. 2021 Mar 4;9:616520. doi: 10.3389/fcell.2021.616520 (PMC7969656; doi:10.3389/fcell.2021.616520)
Supplement: Supplementary file 2 [file Data_Sheet_1.docx]

**
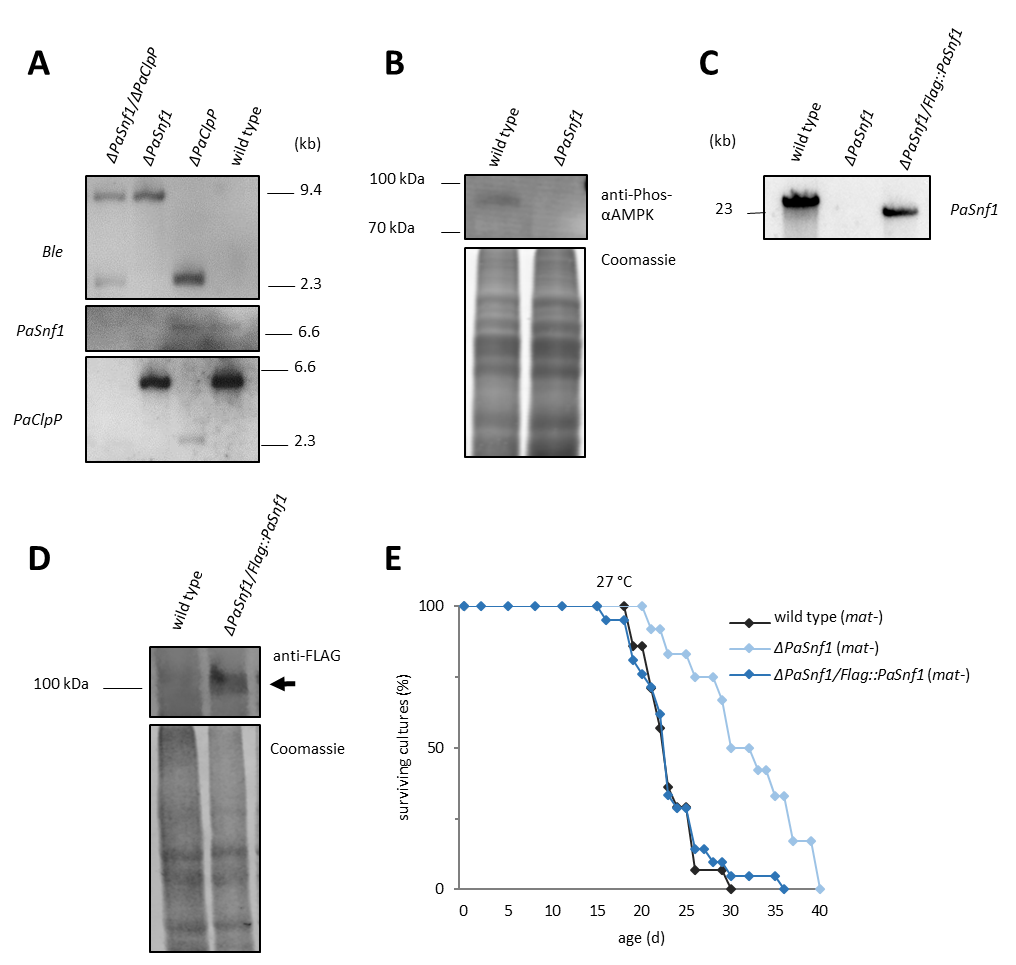
**

**Supplemental FIGURE S1|** Deletion of *PaSnf1* and complementation of *ΔPaSnf1*.

**(A)** Southern blot analysis of *ΔPaClpP*, *ΔPaSnf1* and *ΔPaSnf1*/*ΔPaClpP*. 1 µg DNA was treated with HindIII and separated in an 1 % agarose gel. After transfer to a nylon membrane, the phleomycin resistance cassette (*Ble*), the *PaSnf1* and the *PaClpP* gene were detected with digoxigenin-labeled probes. **(B)** Western blot analysis of 150 µg total protein extracts of *ΔPaSnf1* and wild type with an anti-Phos-αAMPK antibody. A 12 % SDS polyacrylamide gel was used for this analysis. After transfer, the gel was stained with coomassie and serves as loading control. **(C)** Southern blot analysis of *ΔPaSnf1* and *ΔPaSnf1*/*Flag::PaSnf1.* 1 µg DNA was treated with BamHI and separated in a 1 % agarose gel. After transfer to a nylon membrane, the *PaSnf1* gene was detected with a digoxigenin-labeled probe. **(D)** Western blot analysis of 100 µg total protein extracts of *ΔPaSnf1*/*Flag::PaSnf1* and wild type with an anti-FLAG antibody. An 8 % SDS polyacrylamide gel was used for this analysis. After transfer, the gel was stained with coomassie and serves as loading control. **(E)** Lifespan analysis of *ΔPaSnf1* (*mat-*) (n = 12, p < 0.001), *ΔPaSnf1*/*Flag::PaSnf1* (*mat-*) (n = 21, p = 0.784) and wild type (*mat-*) (n = 14), grown on M2 medium at 27 °C and constant light. “(*mat-*)”  represents the mating type “minus” (*rmp1-1*). The p-values of the lifespan curves in comparison to wild type were determined by SPSS with three different statistic tests. A compilation of all p-values can be found in Tab. S3.


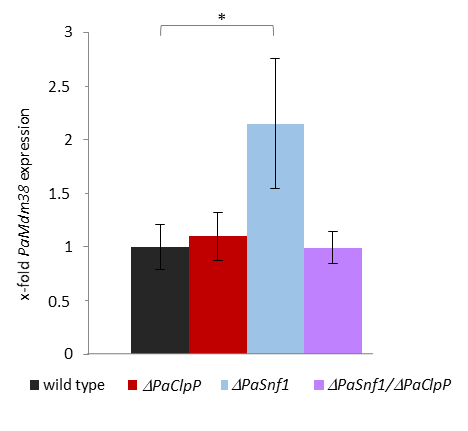


**Supplemental FIGURE S2|** Ablation of PaSNF1 affects *PaMdm38* expression.

Quantitative realtime PCR (qRT-PCR) of *ΔPaSnf1* (n = 5)*, ΔPaClpP* (n = 6)*, ΔPaSnf1*/ *ΔPaClpP* (n = 6) and wild type strains (n = 9), normalization to the *Porin* expression. The expression level of wild type was set to 1. Values presented are mean ± SEM (***: p < 0.001; **: p < 0.01; *p < 0.05, two-tailed Student’s *t*-test).

**Table S2|** Overview of lifespan and growth rate of *P. anserina* strains cultured under different conditions. The p-values were determined with SPSS (IBM SPSS Statistics, New York, USA) with three different tests. Indicated are the p-values of the lifespan curves in comparison to the wild type. “N” refers to the number of analyzed biological replicates. Mating type “plus” and “minus” cultures are combined. “p-value 1” = Log Rank (Mantel-Cox); “p-value 2” = Breslow (Generalized Wilcoxon); “p-value 3” = Tarone-Ware.

|  | wild type | *ΔPaClpP* | *ΔPaSnf1* | *ΔPaSnf1/*  *ΔPaClpP* |
| --- | --- | --- | --- | --- |
| M2 at 27 °C | | | | |
| mean lifespan (d)  ± SEM | 23  ± 0.51 | 68  ± 5.32 | 28  ± 1.22 | >186  ± 34.96 |
| maximum  lifespan (d) | 30 | 144 | 40 | >630 |
| p-value 1 | / | 6.748 E-13 | 1.031 E-4 | 3.815 E-14 |
| p-value 2 | / | 8.961 E-11 | 7.940 E-4 | 5.921 E-12 |
| p-value 3 | / | 7.529 E-12 | 2.822 E-4 | 4.590 E-13 |
| growth rate (cm/d)  ± SEM | 0.62  ± 0.014 | 0.64  ± 0.009 | 0.53  ± 0.009 | 0.53  ± 0.007 |
| N | 32 | 22 | 28 | 25 |
| M2 at 35 °C | | | | |
| mean lifespan (d)  ± SEM | 10  ± 0.38 | 9  ± 0.77 | 10  ± 0.82 | 8  ± 0.82 |
| maximum  lifespan (d) | 13 | 16 | 13 | 16 |
| p-value 1 | / | 1.04 E-1 | 2.938 E-1 | 2.353 E-2 |
| p-value 2 | / | 6.912 E-1 | 5.228 E-1 | 3.39 E-4 |
| p-value 3 | / | 3.16 E-1 | 3.745 E-1 | 2.084 E-3 |
| growth rate (cm/d)  ± SEM | 0.45  ± 0.031 | 0.33  ± 0.029 | 0.25  ± 0.036 | 0.23  ± 0.037 |
| N | 25 | 28 | 17 | 19 |
| M2 – N | | | | |
| mean lifespan (d)  ± SEM | 40  ± 2.27 | 62  ± 3.32 | 7  ± 0.51 | 7  ± 0.44 |
| maximum  lifespan (d) | 59 | 110 | 18 | 9 |
| p-value 1 | / | 2.936 E-7 | 3.480 E-13 | 1.294 E-9 |
| p-value 2 | / | 4.509 E-7 | 5.289 E-11 | 5.246 E-9 |
| p-value 3 | / | 3.225 E-7 | 4.363 E-12 | 2.657 E-9 |
| growth rate (cm/d)  ± SEM | 0.37  ± 0.005 | 0.36  ± 0.005 | 0.32  ± 0.014 | 0.29  ± 0.011 |
| N | 23 | 29 | 28 | 9 |
| M2 + glycerol | | | | |
| mean lifespan (d)  ± SEM | 23  ± 3.74 | 9  ± 0.28 | 10  ± 0.95 | 7  ± 0.50 |
| maximum  lifespan (d) | 40 | 13 | 21 | 9 |
| p-value 1 | / | 3.058 E-5 | 2.972 E-4 | 1.089 E-4 |
| p-value 2 | / | 2.900 E-4 | 1.424 E-3 | 1.478 E-4 |
| p-value 3 | / | 9.838 E-5 | 6.716 E-4 | 1.209 E-4 |
| growth rate (cm/d)  ± SEM | 0.51  ± 0.030 | 0.43  ± 0.008 | 0.55  ± 0.013 | 0.46  ± 0.017 |
| N | 10 | 18 | 19 | 9 |

**Table S3|** Overview of lifespan and growth rate of *P. anserina* strains cultured under different conditions. The p-values were determined with SPSS (IBM SPSS Statistics, New York, USA) with three different tests. “N” refers to the number of analyzed biological replicates. Isolates of the two mating types are separated. “p-value 1” = Log Rank (Mantel-Cox); “p-value 2” = Breslow (Generalized Wilcoxon); “p-value 3” = Tarone-Ware.

|  | mating type „minus“ (*rmp1-1*) | | | | | mating type „plus“ (*rmp1-2*) | | | |
| --- | --- | --- | --- | --- | --- | --- | --- | --- | --- |
|  | **wild type** | ***ΔPaClpP*** | ***ΔPaSnf1*** | ***ΔPaSnf1/***  ***ΔPaClpP*** | ***ΔPaSnf1/***  ***Flag::PaSnf1*** | **wild type** | ***ΔPaClpP*** | ***ΔPaSnf1*** | ***ΔPaSnf1/***  ***ΔPaClpP*** |
| M2 at 27 °C | | | | | | | | | |
| mean lifespan (d) ± SEM | 23  ± 0.81 | 77  ± 8.54 | 32  ± 1.83 | >311  ± 52.71 | 23  ± 0.87 | 22  ±0.64 | 57  ± 3.50 | 25  ± 1.17 | 70  ± 5.81 |
| maximum  lifespan (d) | 30 | 144 | 40 | >611 | 36 | 26 | 72 | 33 | 110 |
| p-value 1 | / | 2.468 E-7 | 2.957 E-4 | 2.468 E-7 | 7.836 E-1 | / | 1.445 E-5 | 3.886 E-2 | 8.038 E-8 |
| p-value 2 | / | 2.683 E-6 | 9.972 E-4 | 6.382 E-6 | 9.865 E-1 | / | 1.275 E-5 | 7.446 E-2 | 8.477 E-7 |
| p-value 3 | / | 8.161 E-7 | 5.136 E-4 | 8.161 E-7 | 9.008 E-1 | / | 4.015 E-6 | 5.395 E-2 | 2.451 E-7 |
| growth rate (cm/d) ± SEM | 0.64  ± 0.015 | 0.63  ± 0.012 | 0.56  ± 0.015 | 0.55  ± 0.006 | 0.61  ± 0.031 | 0.61  ± 0.021 | 0.64  ± 0.015 | 0.50  ± 0.008 | 0.51  ± 0.010 |
| N | 14 | 12 | 12 | 12 | 21 | 18 | 10 | 16 | 13 |
| M2 at 35 °C | | | | | | | | | |
| mean lifespan (d) ± SEM | 10  ± 0.39 | 14  ± 0.39 | 9  ± 1.14 | 9  ± 1.16 | / | 9  ± 0.66 | 6  ± 0.55 | 8  ± 1.17 | 6  ± 0.91 |
| maximum  lifespan (d) | 13 | 16 | 13 | 16 | / | 13 | 12 | 12 | 12 |
| p-value 1 | / | 2.235 E-5 | 4.769 E-1 | 6.582 E-1 | / | / | 2.414 E-2 | 7.208 E-1 | 3.394 E-2 |
| p-value 2 | / | 3.180 E-5 | 9.741 E-1 | 1.391 E-1 | / | / | 5.791 E-3 | 6.307 E-1 | 7.586 E-3 |
| p-value 3 | / | 2.359 E-5 | 7.046 E-1 | 3.084 E-1 | / | / | 9.955 E-3 | 7.079 E-1 | 1.367 E-2 |
| growth rate (cm/d) ± SEM | 0.51  ± 0.037 | 0.47  ± 0.019 | 0.23  ± 0.051 | 0.26  ± 0.051 | / | 0.38  ± 0.045 | 0.18  ± 0.029 | 0.23  ± 0.048 | 0.18  ± 0.054 |
| N | 13 | 11 | 10 | 11 | / | 12 | 17 | 7 | 8 |
| M2 – N | | | | | | | | | |
| mean lifespan (d) ± SEM | 45  ± 2.84 | 57  ± 3.36 | 7  ± 0.75 | 7  ± 0.50 | / | 37  ± 3.13 | 66  ± 5.05 | 6  ± 0.33 | 7  ± 1.00 |
| maximum  lifespan (d) | 59 | 76 | 18 | 9 | / | 52 | 110 | 8 | 9 |
| p-value 1 | / | 1.118 E-2 | 5.694 E-7 | 3.233 E-5 | / | / | 6.999 E-6 | 2.238 E-7 | 1.618 E-5 |
| p-value 2 | / | 1.124 E-2 | 1.226 E-5 | 7.613 E-5 | / | / | 1.370 E-5 | 1.846 E-6 | 2.549 E-5 |
| p-value 3 | / | 1.073 E-2 | 2.771 E-6 | 5.072 E-5 | / | / | 9.412 E-6 | 6.523 E-7 | 2.031 E-5 |
| growth rate (cm/d) ± SEM | 0.38  ± 0.006 | 0.37  ± 0.010 | 0.34  ± 0.019 | 0.3  ± 0.012 | / | 0.36  ± 0.006 | 0.36  ± 0.004 | 0.29  ± 0.016 | 0.27  ± 0.021 |
| N | 10 | 12 | 18 | 6 | / | 13 | 17 | 10 | 3 |
| M2 + glycerol | | | | | | | | | |
| mean lifespan (d) ± SEM | 31  ± 2.83 | 10  ± 0.42 | 11  ± 1.36 | 8  ± 0.67 | / | 11  ± 0.87 | 9  ± 0.25 | 10  ± 1.41 | 6  ± 0 |
| maximum  lifespan (d) | 40 | 11 | 21 | 9 | / | 12 | 9 | 21 | 6 |
| p-value 1 | / | 1.155 E-3 | 1.828 E-4 | 9.111 E-4 | / | / | 2.070 E-2 | 5.174 E-1 | 1.431 E-2 |
| p-value 2 | / | 1.556 E-2 | 8.220 E-4 | 1.517 E-3 | / | / | 2.244 E-2 | 1.947 E-1 | 1.431 E-2 |
| p-value 3 | / | 1.325 E-3 | 3.911 E-4 | 1.152 E-3 | / | / | 2.154 E-2 | 2.792 E-1 | 1.431 E-2 |
| growth rate (cm/d) ± SEM | 0.52  ± 0.045 | 0.44  ± 0.011 | 0.54  ± 0.019 | 0.47  ± 0.023 | / | 0.48  ± 0.037 | 0.43  ± 0.010 | 0.56  ± 0.018 | 0.43  ± 0.017 |
| N | 6 | 6 | 10 | 6 | / | 4 | 12 | 9 | 3 |
